# Gadolinium-Doped Cerium Oxide Nanoparticles Embedded in a 3D-Printed System for Antimicrobial Applications

**DOI:** 10.1021/acsomega.6c03833

**Published:** 2026-08-07

**Authors:** Alexandre Silva Santos, Idejan Padilha Gross, João Paulo Santos de Carvalho, Emanoel José Ferreira da Conceição, Ariane Pandolfo Silveira, Mac-kedson Medeiros Salviano Santos, Diego Sousa-Moura, Caio Vinícius Pires Leal, Heloísa Antoniella Braz-de-Melo, Daniel Oliveira Freire, Daniela Castilho Orsi, Marcilio Cunha-Filho, Marcelo Henrique Sousa, Ricardo Bentes Azevedo, Sônia Nair Báo, Sebastião William da Silva

**Affiliations:** † Optical Spectroscopy Laboratory, Institute of Physics, 28127 University of Brasilia, Brasília, Distrito Federal 70910-900, Brazil; ‡ Laboratory of Nanobiotechnology, Department of Genetics and Morphology, Institute of Biological Sciences, University of Brasilia, Brasilia, Distrito Federal 70910-900, Brazil; § Laboratory of Food, Drug, and Cosmetics (LTMAC), School of Health Sciences, University of Brasilia, Brasília, Distrito Federal 70910-900, Brazil; ∥ Green Nanotechnology Group, University of Brasilia, Brasília, Distrito Federal 72220-900, Brazil; ⊥ Microscopy and Microanalysis Laboratory, Cell Biology Department, Institute of Biological Sciences, University of Brasilia, Brasília, Distrito Federal 70910-900, Brazil; # Quality Control Laboratory, University of Brasília, Campus Ceilândia, Brasília, Distrito Federal 72220-900, Brazil; ∇ Laboratory School, Center for Educational Sciences, Federal University of Santa Catarina, Florianópolis, Santa Catarina 88040-900, Brazil

## Abstract

Gadolinium-doped cerium oxide (GDC) nanoparticles were synthesized, citrate-functionalized, and incorporated into 3D-printed poly­(vinyl alcohol) (PVA) systems for antimicrobial applications. Citrate functionalization reduced the hydrodynamic diameter from ∼600 nm to ∼250 nm and increased the zeta potential magnitude from −15 mV to −35 mV, indicating significantly improved colloidal stability over 30 days. Antimicrobial assays against *Escherichia coli*, *Staphylococcus aureus*, and *Candida albicans* revealed that citrate-coated nanoparticles in suspension achieved comparable inhibition at concentrations up to 2-fold lower than uncoated counterparts. Importantly, after incorporation into 3D-printed PVA matrices, the systems retained ≥80% of the antimicrobial efficacy observed in suspension, with maximum log reductions of 5.3 (≈99.9995% killing) against *S. aureus* at low nanoparticle loadings (0.83 mg/mL). Cytotoxicity assays on HaCaT keratinocytes demonstrated a clear dose-dependent response for citrate-coated nanoparticles (IC_50_ = 3.5 mg/mL, R^2^ = 0.986), whereas uncoated nanoparticles showed irregular behavior. Furthermore, zebrafish embryo toxicity tests further confirmed that citrate coating significantly reduced acute toxicity, with 0% mortality at 100 mg/L for GDC-cit versus 80% for uncoated GDC. These results demonstrate that 3D-printed PVA systems incorporating citrate-functionalized GDC nanoparticles constitute a promising, scalable, and safer-by-design antimicrobial platform. The preservation of antimicrobial activity across distinct physicochemical environments highlights the potential of these systems to bridge the gap between nanomaterial development and practical biomedical applications.

## Graphical abstract

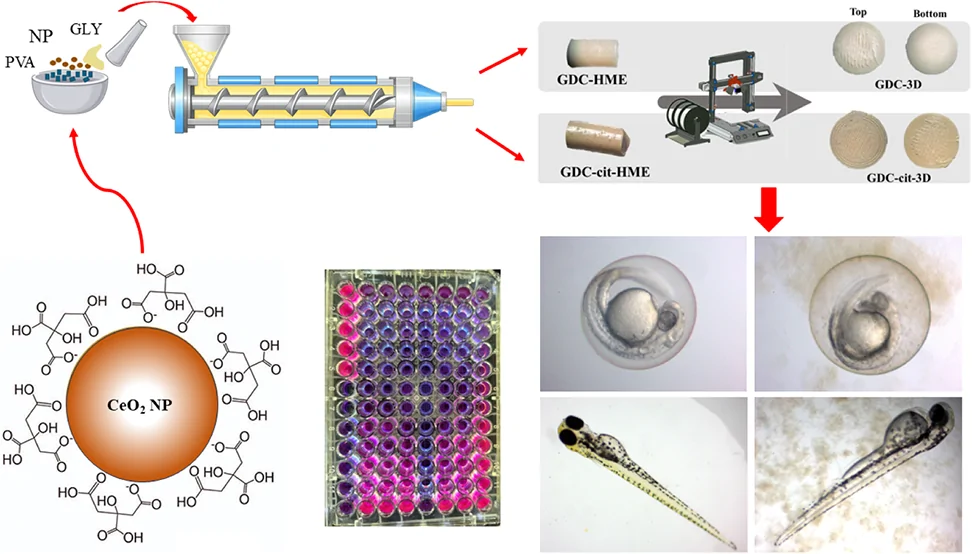

## 1 Introduction

The skin, as the largest organ of the human body, plays a critical role in protection, thermoregulation, sensory perception, and metabolic homeostasis.^1,2^ Despite its protective function, the skin is highly susceptible to a wide range of pathological conditions, including chronic wounds, infections, and inflammatory disorders, which collectively represent a significant global health burden. Recent estimates indicate that skin-related conditions rank among the leading causes of nonfatal diseases worldwide, with chronic wound care alone accounting for annual costs exceeding USD 150 billion.^3,4^

Among these conditions, complications associated with esthetic skin perforations such as earring insertion have received increasing attention. Approximately 35% of individuals who wear earrings experience adverse effects, including infections, allergic reactions, and undesirable scarring.^5^ These complications are frequently linked to metal-induced irritation and microbial contamination, often requiring a therapeutic intervention with anti-inflammatory and antimicrobial agents. In this context, topical drug delivery systems emerge as a particularly attractive strategy as they enable localized treatment while minimizing systemic exposure. By confining the active agent to the outer skin layers, these systems offer improved safety profiles, targeted therapeutic action, and the potential for sustained drug release.^6^

In recent years, three-dimensional (3D) printing technologies have revolutionized the design of pharmaceutical dosage forms, enabling the fabrication of highly customizable and structurally complex systems. Extrusion-based techniques allow the incorporation of bioactive compounds into polymeric matrices, facilitating precise control over geometry, drug loading, and release kinetics.^7^ This level of design flexibility represents a paradigm shift in drug delivery, enabling the development of patient-specific therapies and multifunctional platforms that integrate structural and therapeutic functionalities into a single device.^6^

Among the available additive manufacturing approaches, fused deposition modeling (FDM) stands out due to its operational simplicity, cost-effectiveness, and compatibility with thermoplastic polymers.^7,8^ In FDM, drug-loaded filaments, typically produced via hot-melt extrusion (HME), are deposited layer by layer to form the final structure. Importantly, the physicochemical properties of the filament can be finely tuned during extrusion, allowing for control over mechanical behavior, drug dispersion, and release profiles.^8,9^ However, despite these advantages, the integration of functional nanomaterials into FDM-based systems remains relatively underexplored, particularly in the context of topical antimicrobial applications.

In this regard, cerium oxide nanoparticles (CeO_2_ NPs) have been extensively investigated due to their unique redox cycling between Ce^3+^ and Ce^4+^ states, which enables catalytic activity and modulation of reactive oxygen species (ROS),^10,11^ rendering them particularly promising for antimicrobial and anti-inflammatory applications.^9,11^ These properties underpin their antimicrobial, antioxidant, and anti-inflammatory activities. Importantly, ROS-mediated antibacterial mechanisms involve oxidative stress induction, membrane disruption, and interference with essential cellular processes, such as DNA replication and respiration.

However, the performance of pure CeO_2_ nanoparticles is often limited by particle aggregation, restricted oxygen vacancy concentration, and suboptimal redox kinetics. Several studies have demonstrated that doping with rare-earth elements is an effective strategy to overcome these limitations by increasing oxygen vacancy density and enhancing catalytic performance.^12^ Gadolinium-doped cerium oxide (Gd_*x*_Ce_1–*x*_O_2−δ_) has attracted increasing attention due to its enhanced oxygen vacancy concentration, improved ionic conductivity, and modified electronic structure.

From a biological perspective, the introduction of Gd^3+^ ions into the CeO_2_ lattice generates additional oxygen vacancies to maintain charge neutrality, thereby enhancing the Ce^3+^/Ce^4+^ redox cycling and promoting ROS generation. This mechanism has been directly associated with improved biological activity including antimicrobial and anticancer effects. Recent work by Sarangi et al. (2026)^13^ demonstrated that Gd-doped CeO_2_ nanoparticles exhibit enhanced ROS-mediated cytotoxicity and improved biocompatibility compared to undoped systems, highlighting their potential in biomedical applications. Similarly, earlier studies^14,15^ reported that doped ceria systems exhibit superior antibacterial performance compared to pure CeO_2_, primarily due to increased oxygen vacancy concentration and enhanced ROS generation. These findings consistently indicate that doping plays a critical role in amplifying the ROS-mediated antimicrobial mechanisms.

Taken together, these studies highlight a clear comparative advantage of doped CeO_2_ over its pristine counterpart, particularly in terms of the ROS generation efficiency, charge transfer dynamics, and biological performance. Nevertheless, despite this growing body of evidence, the integration of doped ceria nanoparticles into scalable and structurally controlled platforms, such as 3D-printed systems, remains poorly explored.

In addition, surface functionalization strategies, such as citrate coating, have been shown to improve nanoparticle dispersion, colloidal stability, and interaction with biological environments. However, their role in modulating ROS generation and antimicrobial performance within 3D-printed matrices remains insufficiently understood.

In this context, the present work addresses these critical gaps by developing a 3D-printed topical drug delivery platform that incorporates gadolinium-doped cerium oxide nanoparticles (GDC NPs). The primary innovation of this study lies in the development of an integrated and scalable antimicrobial platform, in which the physicochemical properties of doped ceria nanoparticles are rationally engineered and preserved throughout the 3D-printing fabrication process. Specifically, this work provides a rigorous quantitative comparison between the efficacy of GDC NPs in a liquid suspension versus their performance when embedded in a solid 3D matrix. While the polymer matrix inevitably introduces a physical barrier, we demonstrate that the high surface-to-volume ratio of the engineered nanoparticles maintains a potent antimicrobial action.

Within this framework, Gd doping is employed as a property-enhancement strategy to increase oxygen vacancy concentration and promote ROS-mediated antimicrobial activity, while citrate functionalization acts as a dispersion and interface-engineering tool that improves nanoparticle stability and interaction with the polymeric matrix. These modifications are not standalone contributions but enabling strategies that ensure the successful implementation of the central concept: a multifunctional, 3D-printable nanocomposite platform with retained and tunable biological performance. This integrated approach addresses a critical gap in the literature, where doped ceria nanoparticles have been extensively studied in isolation but have rarely been translated into structurally controlled, application-ready platforms.

To this end, the developed systems were comprehensively characterized using structural, vibrational, and morphological analyses, alongside biological evaluations, including cytotoxicity, antimicrobial activity, and zebrafish embryo toxicity assays. By bridging materials science, nanotechnology, and pharmaceutical engineering, this study advances the state of the art by demonstrating how surface-functionalized cerium-based nanoparticles can be effectively integrated into 3D-printed systems, yielding multifunctional, biocompatible, and application-ready antimicrobial platforms.

## 2 Material and Methods

### 2.1 Synthesis

The synthesis of gadolinium-doped cerium oxide nanoparticles (GDC NPs) was carried out using the homogeneous precipitation method. Initially, 200 mL of Milli-Q water was placed in a reaction vessel, to which cerium nitrate hexahydrate (Ce­(NO_3_)_3_·6H_2_O, 1.87 g) and gadolinium nitrate hexahydrate (Gd­(NO_3_)_3_·6H_2_O, 0.21 g) were added under continuous stirring. Subsequently, 200 mL of absolute ethanol was introduced into the solution. The solution pH was then adjusted to 11 by the dropwise addition of ammonium hydroxide, and the reaction was maintained under magnetic stirring at 4000 rpm and 60 °C for 2 h. For sodium citrate coating, the as-prepared GDC NPs dispersion was used as the starting medium. A citric acid solution (1 mol/L) was prepared by dissolving 10.50 g of citric acid monohydrate (C_6_H_8_O_7_·H_2_O) in 500 mL of distilled water. From this solution, 100 mL was added dropwise to the nanoparticle suspension until the pH reached approximately 4. Subsequently, sodium citrate (Na_3_C_6_H_5_O_7_·H_2_O, 0.21 g), previously dissolved in 100 mL of distilled water, was added to the mixture. The final suspension was maintained under magnetic stirring at 4000 rpm and 60 °C for an additional 30 min, yielding uncoated GDC NPs and sodium citrate-coated GDC nanoparticles. Finally, the uncoated and citrate-coated NPs were washed and redispersed in Milli-Q water using an Ultra-Turrax homogenizer prior to further processing. The resulting colloidal suspensions were designated GDC and GDC-cit, respectively.

### 2.2 Filament Production by HME

The filament used for 3D system fabrication was produced by HME. Initially, both uncoated and citrate-coated NPs were freeze-dried and subsequently blended with the polymer matrix and plasticizer. Poly­(vinyl alcohol) (PVA; Parteck MXP) was purchased from Merck (Darmstadt, Germany), glycerin (GLY) from Dinâmica (São Paulo, Brazil), and the NPs were obtained according to the synthesis procedure described previously. Prior to extrusion, a physical mixture composed of 70% (w/w) PVA, 15% (w/w) GLY, uncoated or coated GDC NPs was manually homogenized using a mortar and pestle. The resulting samples were designated GDC-HME and GDC-cit-HME, respectively. Extrusion was performed using a corotating twin-screw extruder equipped with a 1.8 mm die (HAAKE, MiniCTW, ThermoScientific, Waltham, MA, USA), operated without recirculation and coupled to a filament tractor system (FTR1) fitted with an automatic diameter gauge (Filmaq3D, Curitiba, Brazil). The screw rotation speed (75 rpm), processing temperature (140 °C), and tractor system velocity were adjusted to ensure homogeneous NP dispersion and uniform filament diameter. All extruded filaments were stored in a desiccator containing silica gel under low relative humidity (RH ∼ 2%) until further use.

To investigate the mechanical properties of the filaments, tensile tests were performed until fracture (n = 3). The experiments were conducted using a universal testing machine (Shimadzu EZ Test, Tokyo, Japan) equipped with a 5 kN load cell. Wedge-type grips were used to secure the filaments, which were aligned horizontally prior to testing and elongated vertically during the assay. Tensile tests were carried out at a constant crosshead speed of 10 mm·min^–1^. Filament specimens measured 60 mm in length, with an initial grip separation of 30 mm and a preload force of 1 N applied before testing.

### 2.3 Production of 3D System by FDM

Cylinder-shaped 3D systems, with a diameter of 13 mm and a height of 1.0 mm, were designed using the free version of Tinkercad software (Autodesk Inc., San Rafael, CA, USA) and subsequently sliced using Creality Print 4.3 software. The structure was fabricated using the previously prepared HME filaments on a Creality FDM 3D printer (model CR-10 SE) equipped with a 0.4 mm brass nozzle. The nozzle temperature was set to 180 °C, while the printing bed temperature was maintained at 65 °C throughout the process. The layer height was fixed at 0.2 mm, and a rectilinear infill pattern with a density of 70% was employed. Three contour walls were printed around each object to ensure dimensional stability and surface uniformity. The printing speed was adjusted to 30 mm·s^–1^ for extrusion movements and 50 mm·s^–1^ for nonextrusion travel movements. Accordingly, two types of 3D systems were produced: those containing uncoated GDC NPs (GDC-3D) and those incorporating citrate-coated GDC NPs (GDC-cit-3D). The true density of the samples was determined using an Ultrapyc 5000 micro gas pycnometer (Anton Paar GmbH, Graz, Austria) employing ultrapure nitrogen as the probe gas. Measurements were carried out at 25 °C using the meso cell for all samples. The measurement protocol employed a reference-first flow direction, applying the fine powder mode for freeze-dried nanoparticles and the monolith mode for filaments and 3D-printed systems. Briefly, a calibrated pycnometer of known volume was filled with the nanoparticle suspension, and its mass was measured using an analytical balance under controlled temperature conditions (25 °C). The density was calculated from the ratio between the measured mass and the known volume of the pycnometer. All measurements were performed in triplicate to ensure reproducibility, and the results were reported as average values.

### 2.4 Characterization of Materials

The intensity-weighted average hydrodynamic diameter (Z-average), polydispersity index (PdI), and Zeta potential (ζ) of GDC and GDC-cit samples were determined by dynamic light scattering (DLS) and electrophoretic mobility measurements using the Zetasizer Nano ZS90 (Malvern Instruments Ltd., United Kingdom). The freshly prepared nanoparticle suspensions were analyzed immediately after synthesis and subsequently stored at 4 °C for stability monitoring over time. Colloidal stability was evaluated by monitoring hydrodynamic diameter (D_H_), PdI, and ζ-potential after 7, 15, and 30 days of storage. Although the final materials were obtained in the solid state, the evaluation of colloidal stability in aqueous suspension represents a critical quality attribute, since the biological assays involve NP exposure in liquid media. For all measurements, the NP suspensions (20.0 mg/mL) were diluted 100-fold, and the pH was adjusted to 4.0. Analyses were performed at 25 °C using a scattering angle of 90°. Each measurement was conducted under identical conditions to ensure reproducibility and comparability of the results. For the semiquantitative assessment of elemental composition, 60 μL of GDC and GDC-cit colloidal suspension were deposited onto smooth carbon tape mounted on an aluminum stub and allowed to dry at room temperature for 24 h. After complete drying, the samples were analyzed by energy-dispersive X-ray spectroscopy (EDS) coupled to scanning electron microscopy (SEM), to identify the elements present on the NP surfaces. Following EDS analysis, the samples were sputter-coated with gold using a Leica EM SCD 500 sputter coater (Austria) to enhance surface conductivity. SEM images were subsequently acquired using a JSM-7001F microscope (JEOL, Japan) at various magnifications, depending on the sample, and an accelerating voltage of 15.0 kV. To evaluate the spatial distribution of GDC and GDC-cit NPs within the 3D-printed systems, the samples were sectioned to expose both surface and internal regions, followed by sputter coating prior to SEM/EDS analysis.

X-ray diffraction (XRD) measurements were performed using a Panalytical diffractometer operating in the θ–2θ configuration, over an angular range of 10° ≤ 2θ ≤ 85°, with a step size of 0.05° and a scan speed of 0.5° min^–1^. CuK_α_ radiation (λ = 1.5406 Å) was employed. The diffraction data were refined using the Rietveld method with GSAS software and the EXPOGUI interface, utilizing a crystalline silicon standard (NIST 640d) for instrumental calibration. The background was modeled using a first-type Chebyshev polynomial, and profile function 1 was refined with lattice parameters, zero shift, LX, LY, and isotropic displacement (Uiso) parameters.

The Fourier transform infrared (FTIR) spectra were acquired using a Bruker Vertex 70 spectrometer, operated in the Attenuated Total Reflectance (ATR) configuration. An average of 96 scans was collected for each sample, with a spectral resolution of 4 cm^–1^, over the 400 to 4000 cm^–1^ wavenumber range. Background spectra were recorded prior to each measurement to ensure baseline correction. Micro-Raman spectroscopy was performed using a LabRam HR Evolution spectrometer (HORIBA Scientific), equipped with a confocal microscope, a Charge-Coupled Device (CCD) detector, and an 1800 lines·mm^–1^ diffraction grating. The system employed a 405 nm diode laser with an output power of approximately 5 mW, focused onto the sample using a 50× objective lens.

### 2.5 Biological Tests

Immortalized human keratinocytes (HaCaT cells) were maintained in Dulbecco’s Modified Essential Medium (DMEM) supplemented with 10% fetal bovine serum (FBS) and 1% penicillin-streptomycin, at 37 °C in a humidified atmosphere containing 5% CO_2_. For treatment, cells were seeded into 96-well plates at a density of 5 × 10^4^ cells per well and, after 24 h of incubation, exposed to logarithmically spaced concentration series of GDC and GDC-cit formulation (T1–T22; serial dilution). Control groups included a negative control (culture medium/vehicle) and a positive control for cell death, consisting of 50% dimethyl sulfoxide (DMSO). After 24 h of exposure, Alamar Blue reagent (Invitrogen #A50100) was added at 10% (v/v), and the plates were incubated for an additional 2 h. Fluorescence intensity was measured using the Varioskan LUX Multimode Microplate Reader (Thermo Fisher Scientific, Massachusetts, United States), with an excitation wavelength of 560 nm and an emission wavelength of 590 nm.

The antimicrobial activity of the samples was evaluated using the microdilution method in 96-well plates against *Escherichia coli* (ATCC 25922), *Staphylococcus aureus* (ATCC 29213), and *Candida albicans* (ATCC 90028). Bacterial strains were cultured on blood agar at 36.5 °C for 24 h, whereas *Candida albicans* was cultured on Sabouraud dextrose agar at 35 °C for 48 h. Microbial suspensions were prepared in sterile 0.85% (w/v) sodium chloride solution and adjusted to a turbidity equivalent to a 0.5 McFarland standard (1–1.5 × 10^8^ CFU/mL) using a UV–Vis spectrophotometer (UV-1800, Shimadzu, Kyoto, Japan). Turbidity measurements were performed at 625 nm for bacterial suspensions and at 530 nm for *Candida albicans*. The standardized suspensions were subsequently diluted to obtain final inocula of approximately 1.5 × 10^5^ CFU per well for bacteria and 5.0 × 10^2^ to 2.5 × 10^3^ CFU per well for *Candida albicans* in the microdilution assay.

The 3D-printed samples containing 15% (w/w) of NPs were accurately weighed and dissolved in 2 mL of Mueller–Hinton broth for bacterial assays or RPMI-1640 medium for fungal assays. 2-fold serial dilutions were then prepared to obtain the desired test concentrations. Aliquots of 100 μL of each dilution were dispersed into individual wells, followed by the addition of 100 μL of the corresponding microbial inoculum, resulting in a final volume of 200 μL per well. The plates were incubated at 36.5 °C for 24 h for *Escherichia coli* and *Staphylococcus aureus* or for 48 h in the case of *Candida albicans*. Microbial growth was assessed by measuring absorbance using an ELISA plate reader. Wells exhibiting low or no apparent growth were subsequently plated onto Mueller–Hinton agar (*E. coli*, *S. aureus*) or Sabouraud dextrose agar (*C. albicans*) to confirm growth inhibition. In parallel, 20 μL of resazurin solution was added to each well as a viability indicator, with microbial inhibition confirmed by the absence of a color change. Statistical analysis of microbiological data was performed using the online version of GraphPad. Results were analyzed using an unpaired *t*-test, and the level of statistical significance was set at 0.05.

The Fish Embryo Toxicity (FET) test was conducted in accordance with OECD guideline 236,^16^ with adaptations as described by Sousa-Moura.^17^ Zebrafish (*Danio rerio*) embryos were exposed to different concentrations (0, 1.0, 2.5, 6.3, 15.8, 39.8, and 100 mg/L) of GDC and GDC-cit samples in aqueous solution. Based on previous range-finding assays employing successive dilutions of the stock solutions, a total of eight independent tests were selected for analysis. Each treatment consisted of 60 embryos, distributed into three replicates using 96-well microplates. Twenty wells were filled with 250 μL of the test solution, and four wells contained water as an internal plate control, as required in the OECD guideline. One fertilized egg was placed in each well. Exposure was initiated immediately after fertilization and maintained for 96 h in a climate-controlled chamber (SL-24, Solab Científica, Brazil). Embryos and larvae were monitored daily under a stereomicroscope, with developmental parameters evaluated at 70× magnification for eggs and 40× for hatched larvae. The qualitative assessment encompassed multiple phenotypic markers, such as egg coagulation, otolith formation, general developmental delay, eye and body pigmentation, somite formation, heartbeat, edemas, detachment of the tail-bud from the yolk sac, yolk sac absorption, tail malformation, and hatching rate. Following the embryotoxicity assays, the chorion of embryos exposed to different concentrations of GDC and GDC-cit samples was collected and analyzed by scanning electron microscopy (SEM; JSM-7001F, JEOL, Japan) to identify potential morphological alterations induced by the treatments.

## 3 Results and Discussion

### 3.1 Structural, Morphological, and Vibrational Characterization

As shown in Figure 1a, the extrusion process produced uniform filaments with a relatively smooth surface, suitable for the fabrication of the 3D system. The printed structures exhibited good interlayer adhesion, resulting in well-finished specimens without visible defects. Such filament uniformity is essential to ensure consistent material flow during printing, minimizing the occurrence of clogging and extrusion-related defects that could compromise the final product.^18^ The top and bottom surfaces display the characteristic layered texture associated with the printing process, indicating uniform material deposition and stable extrusion conditions throughout the fabrication.

**Figure 1:**
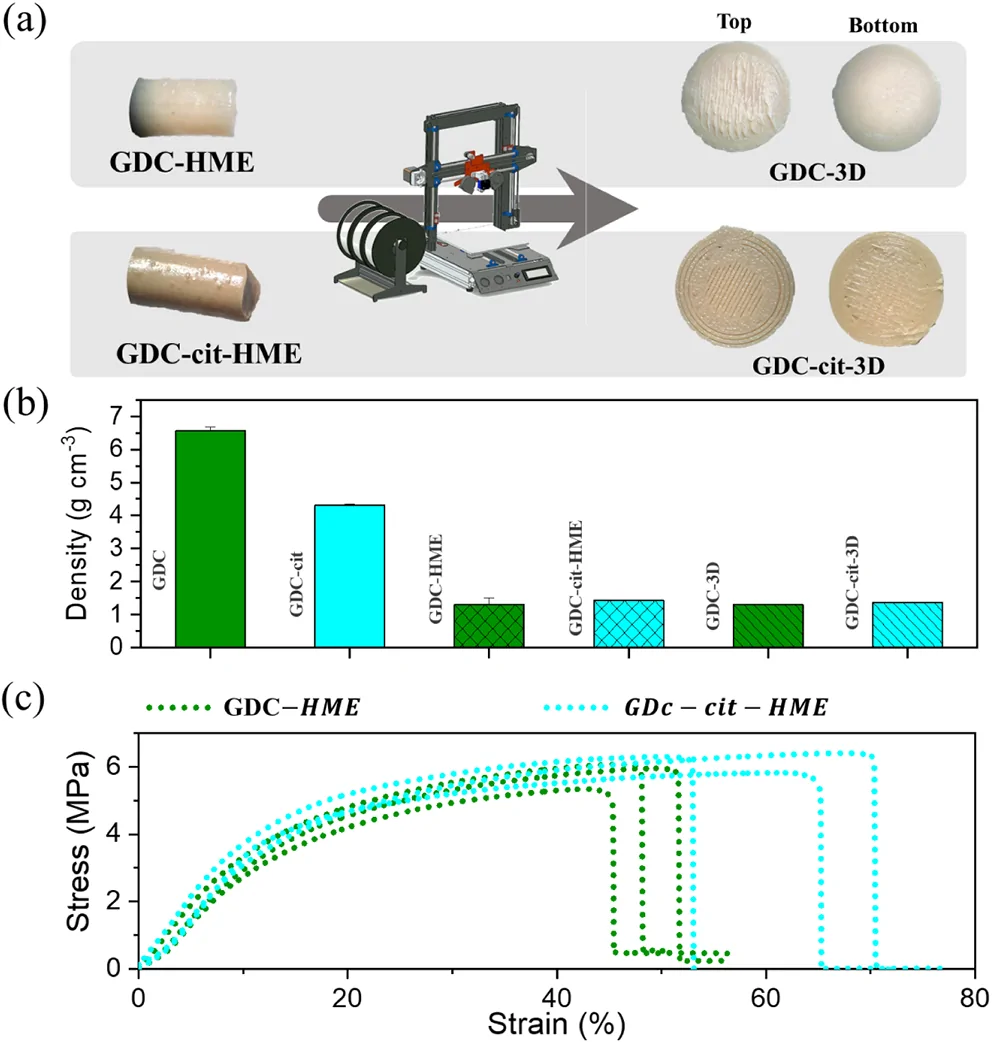
(a) Schematic illustration of the HME-produced filaments (GDC-HME and GDC-cit-HME) and the corresponding FDM-fabricated 3D systems (GDC-3D and GDC-cit-3D), showing top and bottom views. (b) True density values obtained by gas pycnometry (*n* = 3 independent measurements per sample) for freeze-dried nanoparticles, HME filaments, and the corresponding 3D-printed systems. Data are expressed as mean ± standard deviation (SD). (c) Representative stress–strain curves from tensile tests performed on HME filaments (*n* = 3 replicates per condition).

The true density of the NPs (Figure 1b) was, as expected, significantly higher than that of the corresponding filaments and 3D-printed systems, reflecting the highly crystalline nature of the inorganic phase. Theoretical crystallographic densities of gadolinium-doped cerium oxide are typically reported in the range of 7.1–7.2 g/cm^3^.^19–21^ In contrast, the true densities determined in this study for NPs with and without citrate coating were 4.317 ± 0.016 g/cm^3^ and 6.598 ± 0.126 g/cm^3^, respectively, indicating that the presence of the citrate layer substantially reduces the overall density. This reduction is attributed primarily to the markedly lower density of the organic citrate coating compared to the inorganic GDC phase. It is noteworthy that the experimentally measured densities are lower than theoretical crystallographic values, suggesting the presence of closed pores and voids formed during the freeze-drying process. Such features may lead to incomplete densification and internal agglomeration within the nanoparticle powder. In contrast, the true densities of the 3D system were identical to those of the corresponding filaments, indicating chemical and structural stability during the printing process and suggesting that no additional closed porosity was introduced during filament extrusion or FDM manufacturing.

This outcome suggests that the optimization of printing parameters effectively minimizes the formation of voids and interlayer defects, thereby promoting a homogeneous material deposition throughout the printed structure. Previous studies have highlighted that the control of porosity and crystallinity is crucial for improving the mechanical performance of 3D-printed systems. For instance, Liao and collaborators^22^ demonstrated that postprinting heat treatment can enhance mechanical properties by promoting crystallinity and mitigating anisotropy inherent to layer-by-layer fabrication. In addition, nondestructive porosity assessments based on terahertz time-domain spectroscopy have shown that variations in printer settings directly influence density and internal structure, underscoring the importance of precise thermal and processing control to avoid inhomogeneities and structural defects.^23^

The stress–strain curves shown in Figure 1c indicate that filaments containing citrate-coated GDC NPs exhibit higher tenacity and greater elongation at break compared to those incorporating uncoated particles. This behavior may be associated with improved particle dispersion and enhanced interfacial compatibility provided by the citrate coating. The presence of surface carboxylate groups can contribute to electrostatic and steric stabilization, reducing nanoparticle agglomeration and favoring a more homogeneous distribution within the polymer matrix. Such a distribution is expected to limit stress concentration sites and delay crack initiation, thereby facilitating more efficient stress transfer and increased ductility. In addition, the organic coating may function as a compliant interphase between the inorganic surface and the polymer, mitigating mechanical mismatch and contributing to delayed fracture propagation. Indeed, similar effects are well documented in polymer nanocomposites employing surface-modified oxides, where ligand functionalization has been shown to enhance load transfer and toughness.^24,25^

Furthermore, the reproducibility of the mechanical responses among different samples suggests consistency in both the formulation and extrusion processes. Yang et al.^26^ reported that filaments exhibiting breaking stresses above approximately 8.5 MPa are generally suitable for withstanding the traction and rolling forces imposed by FDM printers. Although the filaments produced in the present study displayed lower breaking stress values, no printing difficulties were observed during processing. This behavior may indicate that the flexibility of the material partially compensated for the reduced tensile strength, enabling it to tolerate the mechanical stresses applied during feeding. The observed mechanical compliance likely facilitates stress redistribution and reduces the probability of a brittle fracture during extrusion. As emphasized by Lima et al.,^27^ achieving an adequate balance between tensile strength and viscoelastic behavior is critical for continuous filament feeding and stable printing performance, which appears to be consistent with the results observed in this work.

Figure 2 illustrates the evolution of the hydrodynamic size (Z-average) and zeta potential of GDC and GDC-cit NPs over a 30-day period, providing relevant information regarding their colloidal stability in aqueous media. This evaluation is particularly important since the biological behavior was assessed through suspension-based assays. The GDC NPs (Figure 2a) initially exhibit a relatively high Z-average (∼1500 nm) and polydispersity index (PDI), which decrease significantly within the first 5 days. This behavior suggests a progressive disaggregation process, followed by stabilization at approximately 600 nm, with a relatively constant PDI (∼0.2). In contrast, the GDC-cit NPs (Figure 2b) maintain a considerably smaller Z-average (∼250 nm) with only minor fluctuations over time, and a consistently low PDI (∼0.3), indicating improved colloidal stability associated with citrate functionalization. These results are consistent with previous reports,^28^ which demonstrated that citrate functionalization tends to produce smaller hydrodynamic diameters and more negative zeta potentials. Such surface modification can enhance electrostatic and steric stabilization, thereby influencing nanoparticle dispersion, effective surface availability, and interaction with biological systems in aqueous environments.

**Figure 2:**
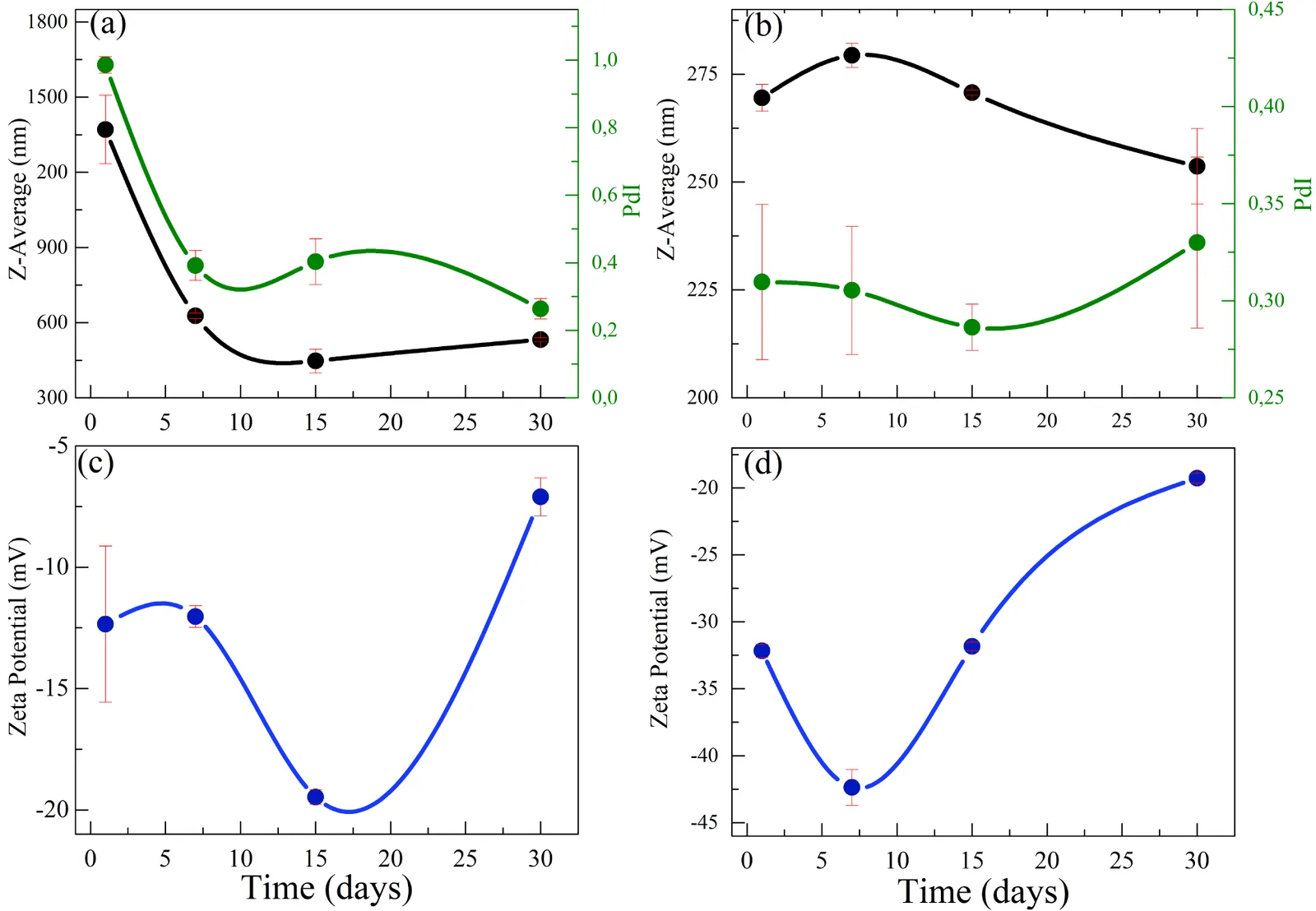
Time evolution of the Z-average hydrodynamic diameter, polydispersity index (PdI), and zeta potential of GDC (a, c) and GDC-cit (b, d) nanoparticles, measured in aqueous suspension during storage at 4 °C for 30 days. Measurements were performed in triplicate (*n* = 3) for every time point, with results reported as mean ± SD.

ζ-potential profiles further corroborate this behavior. GDC NPs (Figure 2c) exhibit a shift from approximately −15 mV to −5 mV over time, indicating a progressive reduction in electrostatic repulsion that may compromise colloidal stability. In contrast, GDC-cit NPs (Figure 2d) display a markedly more negative surface potential, initially around −35 mV and increasing to nearly −20 mV, which supports the role of citrate functionalization in enhancing dispersion stability through increased electrostatic repulsion. This observation is consistent with the findings of Liu et al.,^29^ who demonstrated that surface modifications of GDC NPs, including interactions with redox-active species, significantly influence surface charge and, consequently, suspension stability. Their results indicate that improved hydrophilicity and surface charge density contribute to suppressing uncontrolled aggregation. Collectively, the present results underscore the critical role of surface functionalization in governing the colloidal behavior of cerium oxide-based NPs, particularly under biologically relevant aqueous conditions. Citrate acts as an effective stabilizing agent by combining electrostatic repulsion and steric hindrance, thereby promoting enhanced long-term dispersion stability.

The uncoated (GDC) and citrate-coated (GDC-cit) NPs were morphologically characterized by SEM, and their surface chemical composition was analyzed via EDS. The GDC NPs are presented in Figure 3b and correspond to the colloidal suspension shown in Figure 3a. The SEM micrograph, acquired at 20,000× magnification with a 1 μm scale bar, reveals spheroidal agglomerates with an average size ranging from 50 to 100 nm.

**Figure 3:**
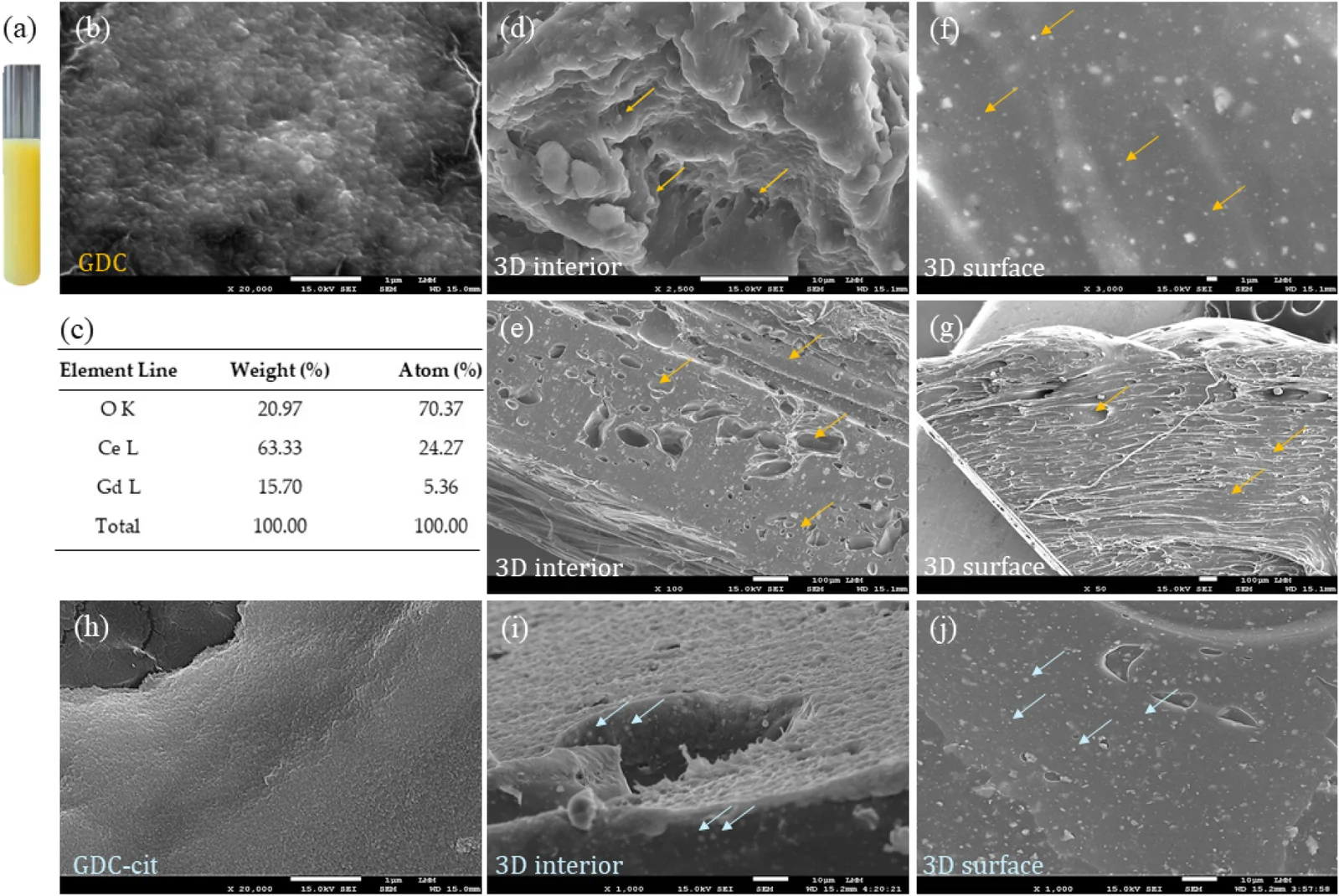
SEM and EDS analysis of NPs and the 3D system of the GDC. (a) Visual appearance of the GDC colloidal suspension and (b) its corresponding SEM micrograph at 20,000× magnification . (c) Quantitative elemental surface composition obtained via EDS data (table) (*n* = 3 analyzed areas). (d) Longitudinal cross-sections showing the distribution of GDC nanoparticles (yellow arrows) in the (d, e) interior and (f, g) surface of the 3D-printed system. Comparative morphology for GDC-cit samples is shown in (h) colloidal form, (i) 3D system interior, and (j) 3D system surface (light blue arrows). All scale bars represent 1 μm for high-magnification micrographs.

For a more detailed structural assessment, the 3D-printer system was longitudinally sectioned, allowing evaluation of both the interior (Figure 3d–e) and the surface regions (Figure 3f–g). The GDC NPs, indicated by the yellow arrows, are clearly discernible within the interior of the 3D-printer system (Figure 3d) and appear even more prominent at the surface (Figure 3f). This observation suggests a homogeneous dispersion of the NPs throughout the 3D matrix rather than a preferential surface segregation.

Its presence of GDC NPs was further confirmed by EDS analysis (table in Figure 3c), which identified oxygen, cerium, and gadolinium with atomic percentages of 70.37%, 24.27%, and 5.36%, respectively, in agreement with the results reported by Swathi.^30^ These findings confirm the successful incorporation and compositional integrity of the nanomaterial within the matrix. The structures observed in Figure 3f range from the nano- to the micrometric scale (based on the 1 μm scale bar), which is likely attributable to particle agglomeration occurring during the printing process.

To evaluate the influence of the citrate coating, printed material samples containing GDC NPs were also subjected to SEM analysis, and their distribution is indicated by the light blue arrows in both the interior (Figure 3i) and surface regions (Figure 3j) of the 3D-printer matrix. Upon comparing the colloidal suspensions of the uncoated (Figure 3b) and citrate-coated nanoparticles (Figure 3h), the coated particles exhibit a discernible surface layer, whereas the uncoated NPs appear more exposed and display greater size heterogeneity. Notably, both micrographs were acquired at the same magnification (20,000×) and share the same scale bar (1 μm), thereby enabling a direct and reliable morphological comparison. Furthermore, the images suggest that the uncoated NPs are considerably larger and more prone to agglomeration. These observations are consistent with the hydrodynamic diameters obtained from Photon Correlation Spectroscopy, which indicate Z-average values of approximately 600 nm (Figure 2a) for GDC and ∼250 nm (Figure 2b) for GDC-cit NPs. The reduction in hydrodynamic size after citrate functionalization reinforces the role of the coating in improving colloidal stability and limiting aggregation.

To investigate the structural and chemical evolution of the nanomaterials’ surface throughout the manufacturing process, we employed X-ray diffraction (XRD), Fourier transform infrared (FTIR), and Raman spectroscopy techniques. XRD was initially used to evaluate the effect of coating on the structural properties of both uncoated and coated GDC NPs. Figure 4a presents the XRD patterns of GDC (i) and GDC-cit (ii) NPs, as well as the corresponding HME-produced filaments (iii and iv), the FDM-fabricated 3D-printed systems containing these NPs (v and vi), and the PVA polymer used in their formulation. The XRD patterns of GDC and GDC-cit NPs (Figure 4a­(i–ii)) can be well indexed to a cubic fluorite-type crystal structure, belonging to the *Fm*3̅*m* space group, in agreement with the crystallographic reference JCPDS 34-0394.^31^ Within the detection limits of the XRD instrument, no secondary crystalline phase was observed, suggesting effective incorporation of Gd^3+^ ions into the ceria lattice. For the filament and 3D-printed samples, the characteristic diffraction peaks of the GDC phase were preserved, indicating that the crystalline structure of the NPs was maintained during the HME and FDM processes. In addition to the reflections associated with the NPs, peaks corresponding to the semicrystalline PVA phase are also observed in the angular range of 10°–50°. These include the diffraction peak of the (100) plane at 11.5°, (101̅) at 19.5°, (101) at 20.1°, (200) at 23.0°, and a composite peak arising from the crystalline planes of (111), (11̅1), (210), and (21̅0) at around 40.5°^32^ (Figure 4a), confirming the coexistence of the polymer matrix and the inorganic phase in the composite systems.

**Figure 4:**
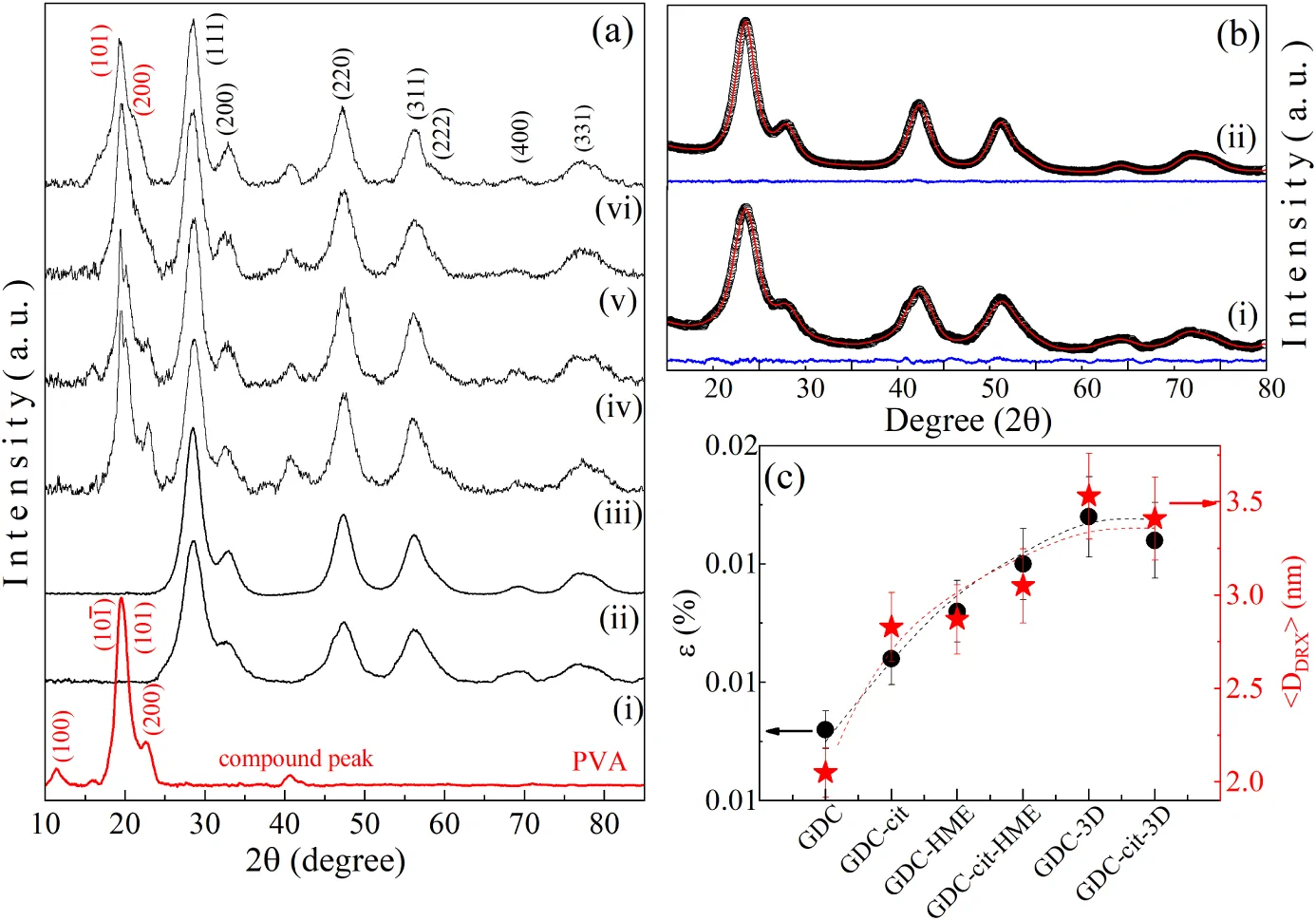
(a) X-ray diffraction (XRD) patterns of the PVA powder and the following samples: (i) GDC, (ii) GDC-cit, (iii) GDC-HME, (iv) GDC-cit-HME, (v) GDC-3D, and (vi) GDC-cit-3D. (b) Rietveld refinement profiles for (i) GDC and (ii) GDC-cit, (ii). (c) Microstrain (ε) and average crystallite size ⟨D_DRX_⟩ for GDC, GDC-cit, GDC-HME, GDC-cit-HME, GDC-3D, and GDC-cit-3D samples from W–H analysis. Error bars: standard errors (size ± 5–8%, strain ± 8–12%). The high quality of linear fits (R^2^ = 0.993–0.996) confirms the statistical reliability of the extracted structural parameters.

To further investigate the effect of surface coating on the X-ray diffraction patterns of all samples, the diffractograms were analyzed using the Rietveld refinement method. Representative refinements for (i) GDC and (ii) GDC-cit NPs are shown in Figure 4b. The results of the Rietveld refinement indicate that the lattice parameter (*a*) remains essentially constant at 5.427 ± 0.002 Å for all samples. This value is significantly higher than that reported for bulk CeO_2_ (4.5411 Å).^33^ This behavior can be explained by considering that the substitution of Ce^4+^ ions by Gd^3+^ ions leads to an expansion of the crystal lattice, which is attributable to the difference in their ionic radii: Ce^4+^ (0.970 Å) and Gd (1.053 Å). Additionally, this ionic size mismatch induces lattice distortions and contributes to the development of microstructural strain within the fluorite structure.

To estimate the lattice microstrain (ε) and the average crystallite size ⟨*D*_*DRX*_⟩ of the GDC NPs for all samples, the Williamson–Hall (W–H) method was employed, in which the broadening of diffraction peaks is correlated with both ⟨*D*_*DRX*_⟩ and ε according to

1 $$\beta \cos\left(\theta \right)=\frac{\lambda k}{\left\langle D_{XRD}\right\rangle }+4\varepsilon \sin\left(\theta \right)$$

In eq 1, λ, β, and *k* represent, respectively, the X-ray wavelength, the full width at half-maximum (fwhm) of the diffraction peaks (corrected using a standard reference sample), and *k* is the shape factor, taken as 0.9 for approximately spherical crystallites. The β-values were extracted from the Rietveld refinement, to ensure high reliability. Following this model, ⟨D_DRX_⟩ is determined from the y-intercept of the β cos­(θ) versus sin­(θ) plot, while ε is obtained from the slope of the linear fit.

The analysis of the W–H plots (Figure 4c) reveals that both the average crystallite size ⟨D_DRX_⟩ and the residual microstrain (ε) increase progressively with the degree of surface coating across all samples.

Before interpreting these trends, it is important to evaluate the applicability and limitations of the W–H method under the present experimental conditions. The W–H approach assumes that peak broadening originates exclusively from the convolution of finite crystallite size (Scherrer-type broadening) and uniform microstrain, while also considering isotropic strain distribution and approximately spherical crystallites. For nanostructured doped oxides such as Gd:CeO_2_, these assumptions may be only partially valid due to factors including anisotropic oxygen-vacancy distributions, dopant-induced local lattice distortions, and possible size–strain correlation.^34^

Nevertheless, the quality of the linear fits (β cos θ versus sin θ) was excellent for all samples, with coefficients of determination ranging from R^2^ = 0.993 to 0.996. These high correlation values indicate that the simplified size–strain model adequately captures the dominant broadening contributions to peak broadening under the investigated conditions, although minor anisotropic or nonuniform effects cannot be completely excluded. The estimated standard errors for ⟨D_DRX_⟩ and ε were ±5–8% and ±8–12%, respectively, supporting the statistical significance of the differences observed between uncoated and citrate-coated samples.

The smaller crystallite size in uncoated GDC NPs (GDC) is consistent with a high density of oxygen vacancies preferentially located at the surface. These defects introduce lattice disorder and structural distortions, which limit the growth of coherent crystalline domains, leading to reduced crystallite sizes in X-ray diffraction analyses. However, alternative explanations cannot be excluded. For example, local compositional fluctuations (inhomogeneous Gd^3+^ incorporation) or nonuniform strain fields arising from vacancy–dopant associations could also contribute to peak broadening in ways that the W–H method partially conflates with size effects. Moreover, the presence of a high density of surface vacancies may induce a tensile surface stress that shifts the effective size–strain balance. In contrast, the increase in ⟨D_XRD_⟩ after citrate functionalization suggests, as a plausible and well-supported hypothesis, that citrate coating and PVA embedding promote the partial passivation of these surface defects, enhancing local lattice ordering, allowing coherent crystalline domains to extend over larger distances, and resulting in an increased average crystallite size for the citrate-coated and polymer-embedded nanoparticles. An alternative, though not mutually exclusive, interpretation is that the citrate layer imposes a compressive surface stress that alters the strain distribution, modifying the W–H fitted parameters without necessarily implying larger defect-free crystallites. High-resolution transmission electron microscopy (HRTEM) or extended X-ray absorption fine structure (EXAFS) would be required to definitively discriminate between these scenarios.

A similar upward trend is observed for residual microstrain (ε). The higher ε values for citrate-coated nanoparticles may arise from either (i) a reduction in oxygen-vacancy-mediated elastic relaxation, which reduces local degrees of freedom for strain accommodation, or (ii) enhanced interfacial stress imposed by the citrate layer and the surrounding PVA matrix. The systematic increase in ε with coating degree, coupled with the excellent fit quality, favors the former interpretation, but the latter cannot be entirely ruled out based on W–H analysis alone.

Figure 5a­(i–vi) presents the FTIR spectra of uncoated (GDC (i)) and citrate-coated (GDC-cit (ii)) CeO_2_NPs, their polymer-containing counterparts (GDC-HME (iii) and GDC-cit-HME (iv)), as well as the final 3D-printed structures (GDC-3D (v) and GDC-cit-3D (vi)).

**Figure 5:**
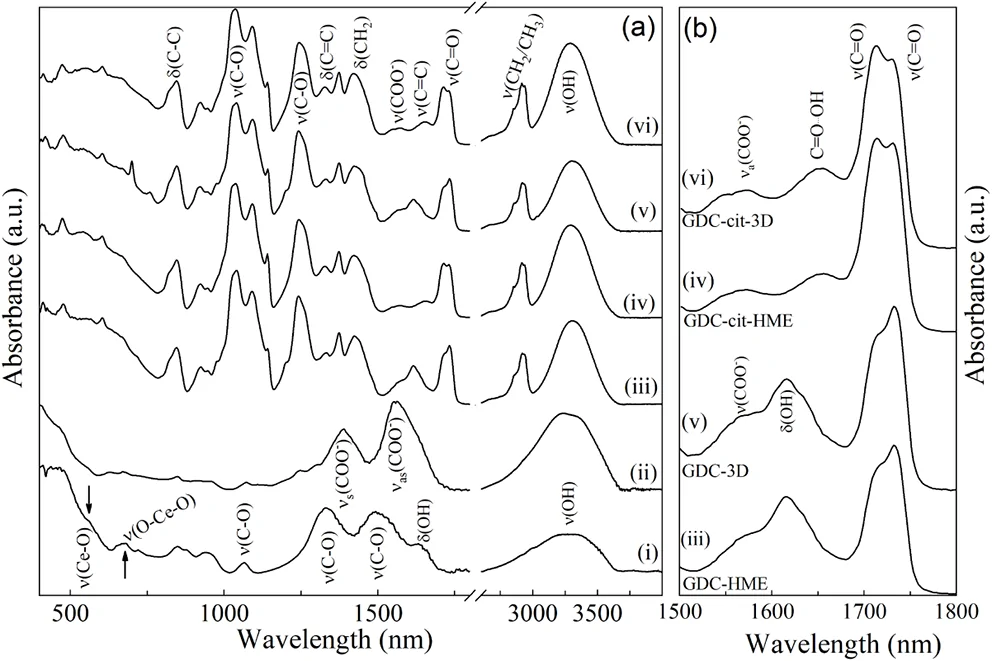
(a) FTIR spectra of (i) GDC, (ii) GDC-cit, (iii) GDC-HME, (iv) GDC-cit-HME, (v) GDC-3D, and (vi) GDC-cit-3D. (b) Magnified view of the FTIR spectra in the 1500–1800 cm^–1^ region for the polymer-containing samples: GDC-HME (iii), GDC-3D (v), GDC-cit-HME (iv), and GDC-cit-3D (vi). All spectra were collected with 96 scans at 4 cm^–1^ resolution. The spectra are representative of *n* = 3 independent sample preparations.

Analysis of the pristine nanoparticles (Figure 5a­(i–ii)) confirms the successful synthesis of crystalline CeO_2_. The broad absorption band between 400–630 cm^–1^ is attributed to Ce–O stretching vibrations, while the feature near 670 cm^–1^ correspond to O–Ce–O lattice vibrations. The band at 850 cm^–1^ ascribed to a phonon envelopment further corroborates the formation of crystalline CeO_2_ NPs.^35^ The broad bands at ∼3300 cm^–1^ and ∼1625 cm^–1^ arise from the O–H stretching and bending vibrations of adsorbed water molecules, respectively.

These spectral differences reveal distinct surface chemistries for the uncoated and citrate-coated NPs. In the uncoated CeO_2_ NPs, bands at 1340 and 1500 cm^–1^ indicate the formation of carbonate-like species on the CeO_2_ surface.^35,36^ In contrast, the spectrum of citrate-coated CeO_2_ NPs is dominated by new features at 1400 and 1560 cm^–1^, which are assigned to the asymmetric (ν_*as*_(COO^–^)) and symmetric (ν*_s_* (COO^–^)) stanching modes of citrate carboxylate groups, respectively.^37^ This spectral feature provides strong evidence for the bidentate chelation of surface Ce^3+^/Ce^4+^ ions by the terminal carboxylate groups of citric acid.

The spectra of the polymer-containing samples (GDC-HME (iii), GDC-cit-HME (iv), GDC-3D (v), and GDC-cit-3D (vi) in Figure 5a­(iii–vi)) are, as expected, dominated by the vibrational modes of the PVA matrix. The high polymer-to-filler ratio (70 wt % PVA, 15 wt % glycerin, 15 wt % GDC NPs) results in intense polymer absorptions that effectively mask the Ce–O vibrations from the ceria core. The PVA spectrum is characterized by a broad O–H stretching band centered at ∼3500 cm^–1^, C–H stretching mode of methylene groups (2800–3000 cm^–1^), and a carbonyl (C=O) stretching band near 1700–1750 cm^–1^ from residual acetate groups, indicative of the polymer’s degree of hydrolysis.^38^ The fingerprint region (1000–1500 cm^–1^) contains complex features from C–O, C–C, and CH_2_ vibrations, with a notable narrow peak at 1145 cm^–1^ correlating with the crystalline fraction of PVA.^39^ Finally, C–C skeletal vibrations are detectable near 850 cm^–1^.

Despite the overall similarity imposed by the polymer matrix, a detailed examination of the 1500–1800 cm^–1^ region (Figure 5b) reveals significant differences that report directly on nanoparticle–polymer interfacial interactions. This spectral window contains overlapping contributions from PVA carbonyl groups (∼1730 cm^–1^), adsorbed water (∼1620–1650 cm^–1^), citrate carboxylates (∼1550–1600 cm^–1^), and surface carbonates. In composites containing uncoated NPs (GDC-HME (iii) and GDC-3D (v)), the band at ∼1730 cm^–1^ (free PVA carbonyls) dominates, and the feature near 1620 cm^–1^, attributed to adsorbed water and carbonates on the hydroxylated CeO_2_ surface, is more intense. Conversely, for citrate-coated systems (GDC-cit-HME (iv) and GDC-cit-3D (vi)), we observe the emergence of weak bands at ∼1560 cm^–1^ (coordinated COO^–^) and a shift of the water-related band to ∼1650 cm^–1^. This shift indicates stronger hydrogen bonding and enhanced water structuring due to the hydrophilic citrate layer. Most importantly, the relative intensity of the carbonyl bands inverts: the ∼1711 cm^–1^ band, consistent with coordinated COO^–^ groups and hydrogen-bonded carbonyls (C=O···OH), becomes more prominent in the citrate-coated samples at the expense of the free carbonyl band at ∼1730 cm^–1^. This spectral evolution strongly suggests that citrate functionalization enhances nanoparticle–polymer compatibility by promoting hydrogen bonding and interfacial coordination while partially passivating surface oxygen vacancies.

To overcome the masking effect observed in FTIR and to directly probe the nanoparticle core and surface after incorporation into the polymer, we employed Raman spectroscopy with a 405 nm excitation wavelength. This wavelength is particularly surface-sensitive, as both incident and scattered radiation are absorbed predominantly within the near-surface region of the CeO_2_ nanoparticles.

Figure 6a displays the Raman spectra of the same sample series (GDC (i), GDC-cit (ii), GDC-HME (iii), GDC-cit-HME (iv), GDC-3D (v), and GDC-cit-3D (vi)). The spectra of the pristine NPs (Figure 6a­(i–ii)) are characterized by the intense F_2g_ vibrational mode of the fluorite structure at ∼460 cm^–1^.^40^ Additional defect-induced modes are observed at ∼550 cm^–1^ and ∼595 cm^–1^, corresponding to extrinsic $\left(V_{O}^{ext}\right)$ and intrinsic $\left(V_{O}^{int}\right)$ oxygen vacancies, respectively. A band at ∼830 cm^–1^ is attributed to peroxide-like species (O_2_^2 –^) adsorbed on surface defects,^41^ while the feature at ∼1173 cm^–1^ originates from a second-order longitudinal optical (2LO) mode.^42^

**Figure 6:**
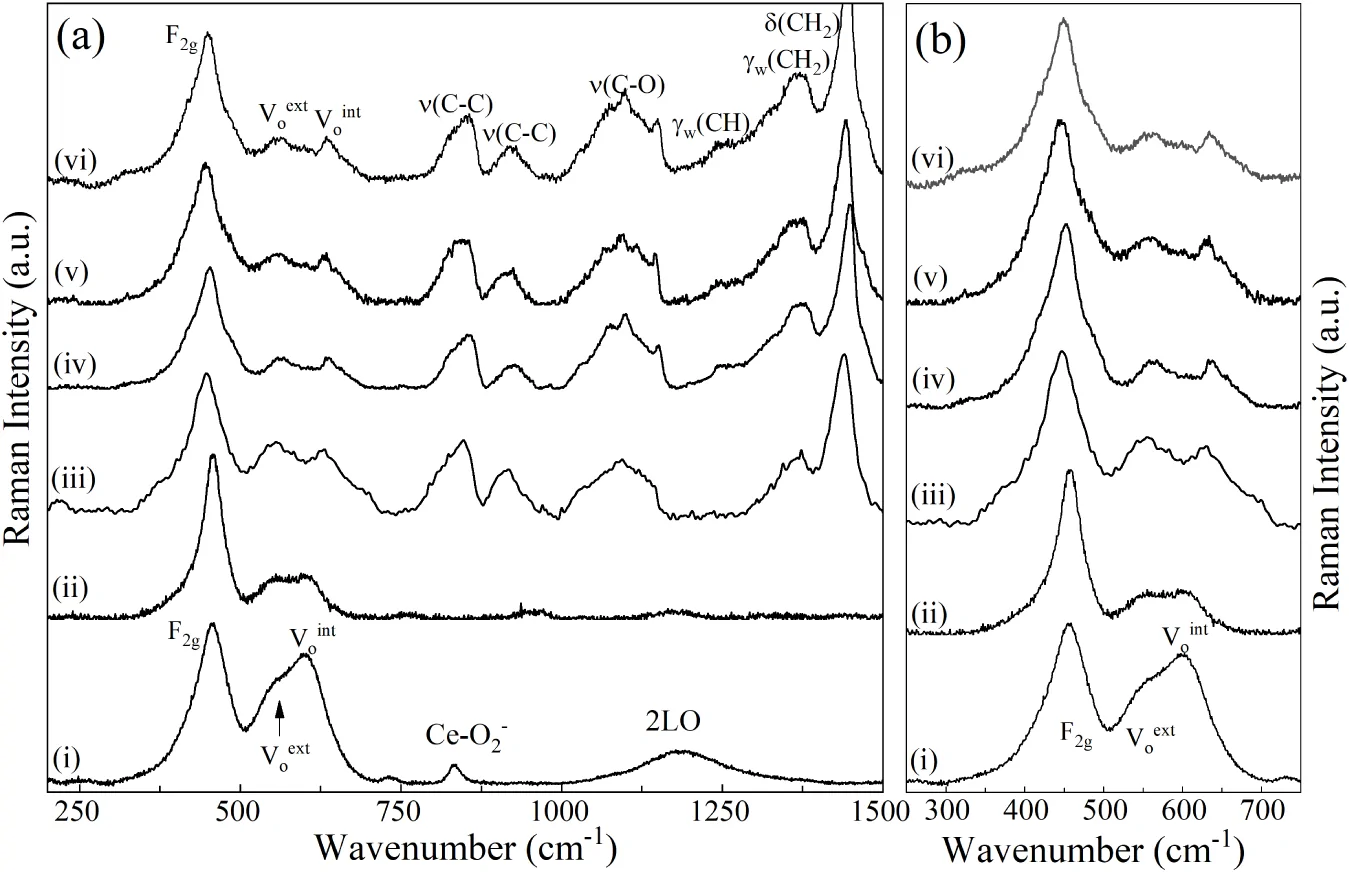
(a) Raman spectra (λ_exc_ = 405 nm) of (i) GDC, (ii) GDC-cit, (iii) GDC-HME, (iv) GDC-cit-HME, (v) GDC-3D, and (vi) GDC-cit-3D. (b) Enlarged view of the Raman spectra in the 250–750 cm^–1^ region, highlighting the evolution of the F_2g_ phonon and defect-induced vacancy modes. Spectra are representative of *n* = 3 measurements per sample.

Notably, the Raman spectra of the polymer-containing samples (GDC-HME (iii), GDC-cit-HME (iv), GDC-3D (v), and GDC-cit-3D (vi)) exhibit a clear superposition of the CeO_2_ NP signals and vibrational bands from the PVA matrix. The persistence of the characteristic cerium oxide peaks after extrusion and 3D printing provides direct confirmation that the structural integrity of the CeO_2_ NPs is preserved throughout the entire fabrication process.

Beyond confirming nanoparticle retention, the Raman spectra reveal subtle but significant modifications in the nanoparticle’s surface structure due to polymer interaction. An enlarged view of the key 250–750 cm^–1^ region (Figure 6b) highlights changes in the oxygen vacancy bands. Compared to the pristine NPs, the composites show a pronounced reduction in the intensity of both the extrinsic (∼550 cm^–1^) and intrinsic (∼600 cm^–1^) oxygen vacancy modes. This attenuation is more pronounced in the citrate-coated samples, directly supporting the FTIR-based hypothesis that interfacial interactions partially passivate surface defects. Furthermore, while the energy of the extrinsic vacancy mode remains largely unchanged, the F_2g_ phonon mode exhibits a noticeable red shift, and the intrinsic oxygen vacancy mode displays a complementary blue shift. These spectral shifts are consistent with alterations in local lattice strain and electronic environment resulting from hydrogen bonding and coordination with the PVA matrix.

In summary, the integration of XRD, FTIR, and Raman spectroscopic analyses provides a comprehensive understanding of the structural and interfacial properties of synthesized nanocomposites. XRD results indicate that the average crystallite size (D_XRD_) and residual strain of the GDC NPs increase progressively with the degree of coating, suggesting that the functionalization process influences the structural integrity and surface energy of the NPs. FTIR spectroscopy further elucidates the surface chemistry, clearly distinguishing between the carbonate-rich surface of uncoated NPs and the carboxylate-terminated surface of citrate-coated NPs. Upon incorporation into the PVA matrix, the FTIR data in the carbonyl region demonstrate that citrate-functionalized NPs establish stronger hydrogen-bonding interactions and coordination with the polymer than their uncoated counterparts. Raman spectroscopy complements these findings by confirming the structural stability of the NPs after processing and providing evidence for the partial passivation of surface oxygen vacancies, an effect that is more pronounced in the case of citrate-coated NPs. Taken together, these multitechnique results demonstrate that the increase in crystallite size and surface strain observed by XRD, combined with citrate-induced chemical compatibility, promotes enhanced interfacial interaction between the NPs and the polymer matrix. This synergy is essential for producing mechanically robust and structurally stable 3D-printed nanocomposite systems with optimized filler–matrix integration.

### 3.2 Biological Tests: Fluorometric Cytotoxicity Assay by Resazurin (Alamar Blue), Antimicrobial Activity, and Fish Embryo Toxicity (FET)

Figure 7a–b presents the results of the cell viability assays performed using HaCaT cells, an immortalized human keratinocyte lineage. This cell line was selected because keratinocytes constitute the predominant cell type of the epidermis and, therefore, represent the first relevant biological interface expected to interact with the nanoparticles during topical applications.^43^ Nevertheless, it is important to recognize the inherent limitations associated with two-dimensional monoculture models. Although HaCaT cells provide a relevant and widely used platform for preliminary cytotoxicity assessment in topical systems, such models do not reproduce the structural complexity, multicellular organization, immune interactions, or barrier properties of the native skin tissue. Consequently, the present results should be interpreted primarily as a comparative *in vitro* evaluation of nanoparticle surface effects rather than as a direct predictor of clinical safety or biological performance *in vivo*.

**Figure 7:**
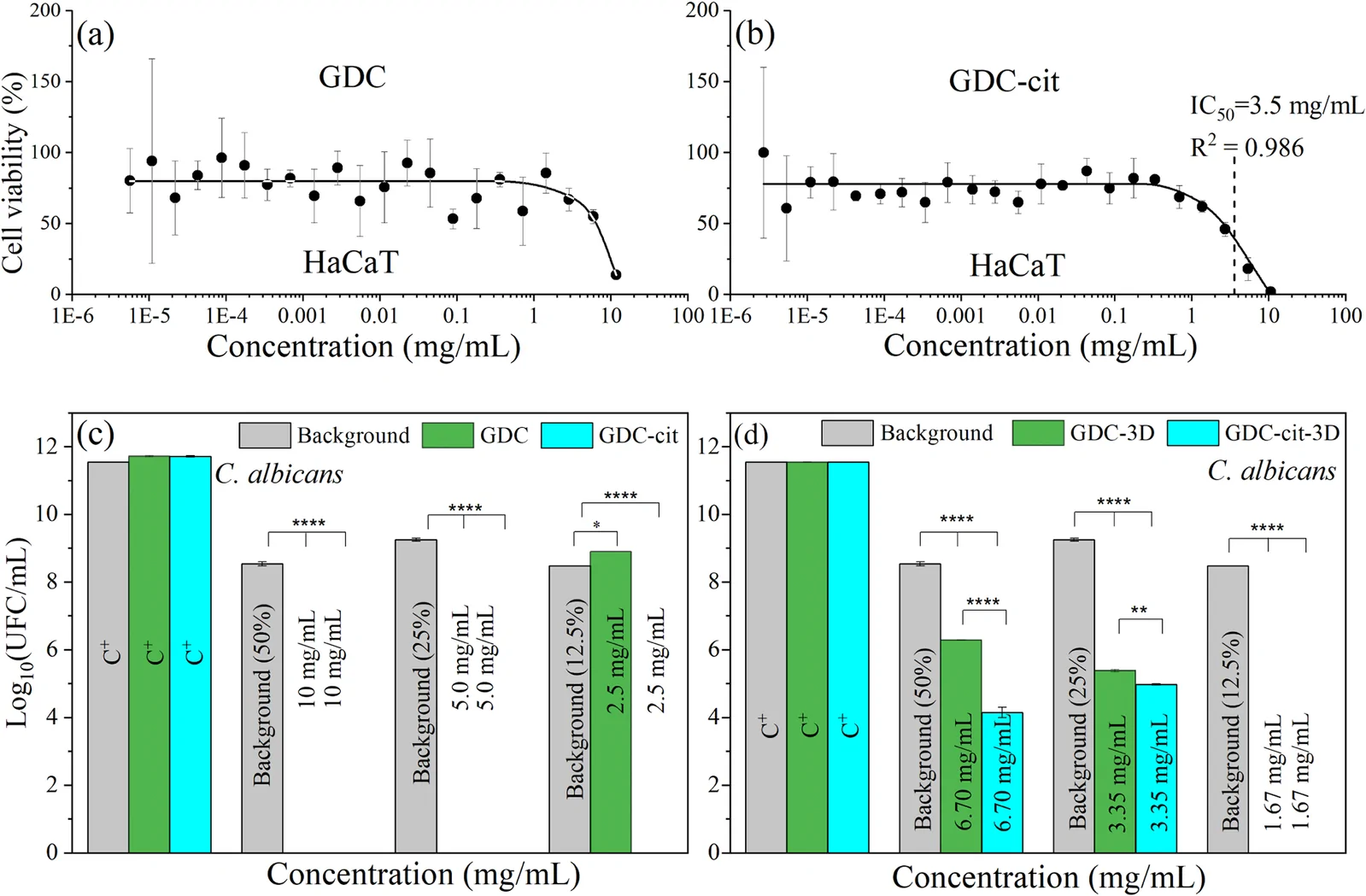
Biological evaluation of nanoparticles using the Alamar Blue cytotoxicity assay and antimicrobial activity tests. In panels (a) and (b), the Alamar Blue assay shows the relative cell viability of immortalized human keratinocytes (HaCaT) after 24 h of nanoparticle exposure to GDC and GDC-cit samples, respectively. Cells were seeded into 96-well plates at a density of 5 × 10^4^ cells per well and, after 24 h, of incubation, exposed to logarithmically spaced concentration series of GDC and GDC-cit formulations (T1–T22; serial dilution). Panels (c) and (d) present the antimicrobial activity against *Candida albicans* for the nanoparticle suspensions and the corresponding 3D systems, including both uncoated and citrate-coated formulations, respectively. In the bar graphs, the gray bars represent the background signal (formulation without nanoparticles), the green bars correspond to GDC, and the blue bars represent GDC-cit. The concentrations are reported as mg of active agent per mL of vehicle, whereas the background group, composed only of the nanoparticle-free excipient matrix, is indicated according to the corresponding 2-fold serial dilution of the formulation (50.0%, 25.0%, 12.5%). Data are mean ± SD (*n* = 6 technical replicates per concentration, from three independent experiments). Statistical analysis: one-way ANOVA with Tukey’s post hoc test. Asterisks indicate significant differences as follows: ****­(*p* < 0.0001), **­(*p* < 0.005), and *­(*p* < 0.05).

Furthermore, it is important to emphasize that two-dimensional monoculture systems cannot fully reproduce the physiological complexity of the epidermis and, therefore, do not allow direct extrapolation to therapeutic dosage conditions. Accordingly, the experiments were specifically designed to evaluate how citrate functionalization influences the interaction between the nanoparticles and epidermal cells.

For both formulations, high cell viability was maintained at low and intermediate concentrations, while cytotoxicity effects became evident only at high concentrations. The GDC-cit formulation (Figure 7b) exhibited a clear dose-dependent behavior, enabling reliable curve fitting and subsequent estimation of the IC_50_ value (3.5 mg/mL, R^2^ = 0.986) with an associated confidence interval. In contrast, the uncoated CeO_2_ NPs (GDC) showed a more variable and nonmonotonic response profile, preventing the determination of a robust and reproducible IC_50_ value.

According to Shinohara et al.,^44^ citrate coating can increase the magnitude of the zeta potential of nanoparticles, thereby enhancing electrostatic repulsion between particles and improving colloidal stabilization, which reduces aggregation and prevents particle collapse. Consequently, citrate-coated nanoparticles tend to exhibit reduced aggregation, more homogeneous dispersion, and more predictable interactions with biological components, such as cells and proteins, which may contribute to lower apparent toxicity and improved reproducibility in biological assays.

The behavior observed in the present study is consistent with this interpretation, as the GDC-cit nanoparticle formulation exhibits a more stable and dose-dependent cell viability profile compared to the uncoated CeO_2_ NPs (GDC), whose biological response is irregular and lacks a clear concentration-dependent trend. These findings support the hypothesis that citrate functionalization enhances the operational biocompatibility of the nanoparticles, not necessarily by reducing their intrinsic biological activity but rather through physicochemical modulation of the nanoparticle interface (e.g., a more negative surface charge and a lower polydispersity index, PDI). Such effects are likely to promote a more uniform and predictable effective exposure of cells to nanomaterials.

From a safety perspective, it is important to emphasize that gadolinium is structurally incorporated into the ceria lattice (Gd_*x*_Ce_1–*x*_O_2−δ_), rather than being present as free Gd^3+^ ions in solution. This structural incorporation reduces the likelihood of ion release under the experimental conditions and contributes to the observed biocompatibility profile. Therefore, the biological responses reported here are primarily governed by surface-mediated interactions of the nanoparticles, rather than by the presence of free gadolinium species.

Figure 7c–d shows the antifungal activity of GDC and GDC-cit NPs evaluated against *Candida albicans* in both colloidal suspension and a 3D system. The results reveal a clear influence of nanoparticle concentration and surface functionalization on the antifungal performance, highlighting the importance of physicochemical control over nanoparticle aggregation and surface reactivity. At equivalent concentrations, both nanoparticle formulations (GDC and GDC-cit) exhibit statistically significant differences relative to the nanoparticle-free background (*p* < 0.0001). Moreover, statistically significant differences were also observed between the two nanoparticle formulations, indicating that GDC-cit NPs are more effective in reducing *C. albicans* viability than uncoated GDC nanoparticles.

For nanoparticles in colloidal suspension, a typical dose-dependent antifungal effect is observed, where increasing nanoparticle concentration leads to a progressive reduction in fungal colony-forming units (CFUs). Notably, the GDC-cit formulation exhibits significantly greater antifungal efficacy than uncoated GDC nanoparticles, as the inhibition of fungal growth is observed even at lower concentrations. Consistent with the behavior observed for antibacterial activity, this enhanced performance is likely attributed to the presence of citrate on the nanoparticle surface, which increases the electrostatic repulsion between particles, thereby preventing agglomeration and maintaining a more stable colloidal dispersion. Such improved dispersion likely results in greater accessibility of reactive surface sites and, consequently, enhanced antifungal activity. Although citrate functionalization was primarily interpreted in terms of improved dispersion and interfacial compatibility, its potential intrinsic biological effects cannot be fully excluded.

The combination of Raman-derived oxygen vacancy signatures, FTIR evidence of surface modification, and colloidal stability data (ζ-potential and DLS) provides consistent indirect support for a surface-mediated redox mechanism. In this context, the antimicrobial activity of cerium oxide nanoparticles is primarily associated with Ce^3+^/Ce^4+^ cycling and the presence of oxygen vacancies, which promote the generation of reactive oxygen species at the nanoparticle interface. The enhanced biological performance observed for citrate-functionalized nanoparticles is therefore attributed to improved surface accessibility and dispersion, which facilitate these interfacial redox processes.

When incorporated into the 3D system, however, a counterintuitive inverse relationship between nanoparticle concentration and antimicrobial activity emerged, whereby higher nanoparticle loadings resulted in a reduced inhibition of *C. albicans*. This behavior is likely associated with confinement-induced aggregation effects within the polymer matrix, which reduce the effective surface area available for interaction with microbial cells. As nanoparticle concentration increases, the formation of larger agglomerates limits the accessibility of oxygen vacancy-rich active sites involved in Ce^3+^/Ce^4+^ redox cycling and ROS generation, thereby partially compromising ROS-mediated fungal damage. In contrast, lower nanoparticle concentrations favor a more homogeneous distribution throughout the printed structure, promoting improved dispersion and greater exposure of reactive surface sites to the surrounding medium, which facilitates antifungal activity. Importantly, this inverse concentration-dependent trend does not indicate a loss of intrinsic nanoparticle functionality, since the same formulations in suspension exhibit a conventional dose-dependent response, confirming that their redox-active antimicrobial properties remain preserved.

Importantly, the incorporation of nanoparticles into the 3D-printed polymeric matrix introduces an additional level of physical confinement, which is expected to reduce direct biological exposure and further mitigate potential risks associated with nanoparticle release or leaching. This aspect is particularly relevant when considering the safe implementation of Gd-doped systems for practical applications.

These findings are consistent with the behavior observed for *E. coli* (Figure 8d), suggesting that excessive nanoparticle density within the matrix promotes agglomerate formation, which limits the accessibility of surface oxygen vacancies and, consequently, reduces the redox processes for microbial inhibition.

**Figure 8:**
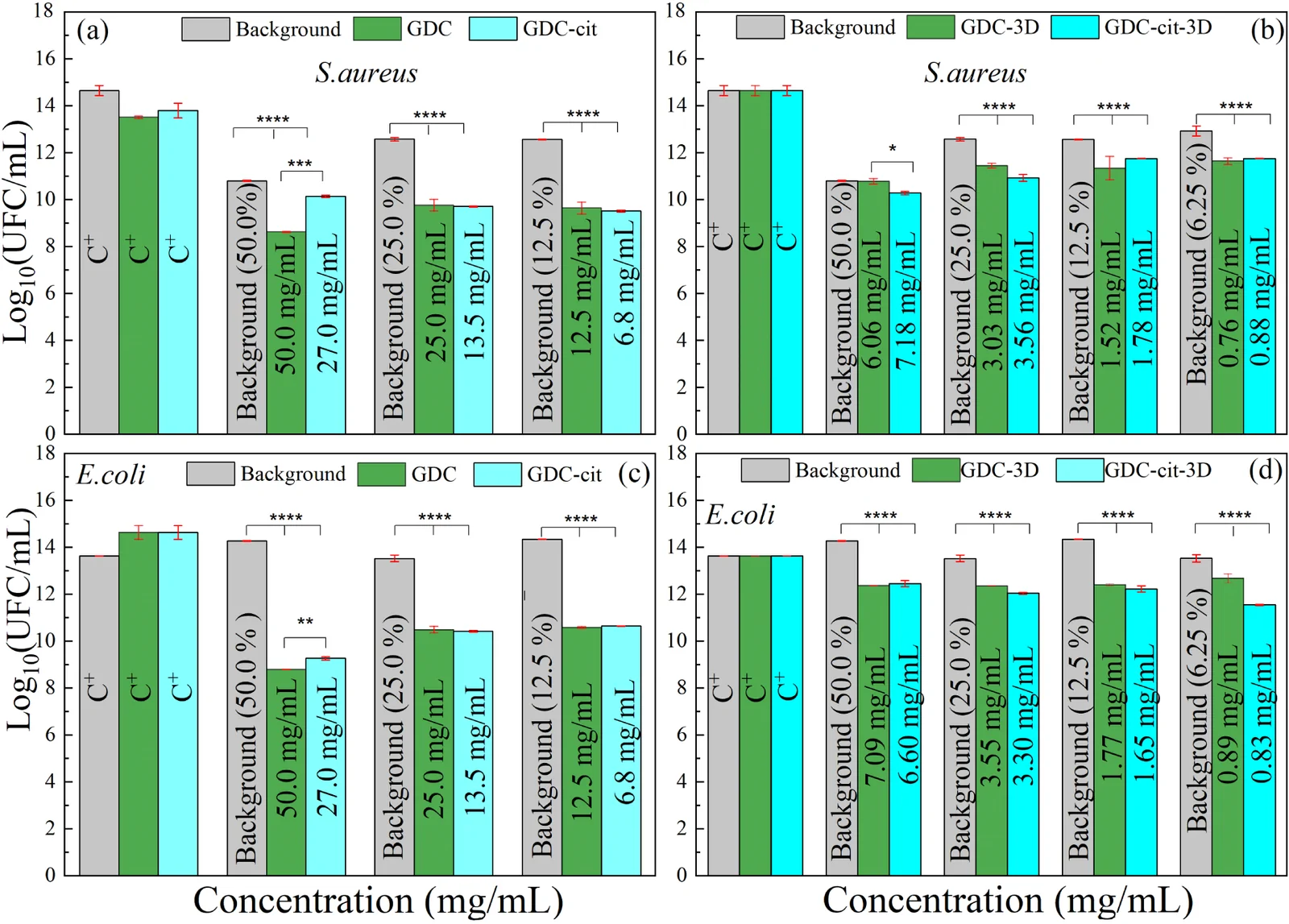
Antimicrobial activity of GDC (green bars) and GDC-cit (blue bars) formulations against *S. aureus* (Gram-positive) and *E. coli* (Gram-negative). Gray bars indicate the background (nanoparticle-free control). (a) *S. aureus*, nanoparticles in suspension; (b) *S. aureus*, 3D system (GDC-3D and GDC-cit-3D); (c) *E. coli*, nanoparticles in suspension; (d) *E. coli*, 3D system. Each assay was performed in triplicate across independent experiments (*n* = 3 independent experiments, each with 3 technical replicates). Exact nanoparticle concentrations (mg/mL) are indicated on the colored bars. Data are mean ± SD (*n* = 9 technical replicates per concentration, from three independent experiments). Statistical analysis: one-way ANOVA with Tukey’s post hoc test. Asterisks indicate significant differences as follows: ****­(*p* < 0.0001), ***­(*p* < 0.0005), **­(*p* < 0.005), and *­(*p* < 0.05).

Nevertheless, even within the 3D system, citrate functionalization significantly enhanced the antifungal performance. The GDC-cit formulation demonstrates effective fungal inhibition at lower concentrations compared to the GDC formulation, confirming that the citrate shell plays a critical role in modulating nanoparticle dispersion, surface activity, and ultimately antifungal activity.

Figure 8 shows that the antimicrobial assays demonstrate concentration-dependent behavior for both *S. aureus* (Gram-positive) (Figure 8a) and *E. coli* (Gram-negative) (Figure 8c) when exposed to the GDC formulation, either with or without sodium citrate coating. At matched concentrations, both GDC and GDC-cit formulations produced a highly significant reduction in bacterial burden for both bacterial strains compared with the nanoparticle-free background for both *S. aureus* and *E. coli* in suspension (*p* < 0.0001). However, unlike the antifungal assays against *C. albicans* (Figure 7c–d), the antibacterial assays against *S. aureus* and *E. coli* (Figure 8a–d) exhibited only limited and less consistent statistical differences between GDC and GDC-cit formulations. This observation suggests that citrate functionalization provides a clearer advantage for antifungal activity than for antibacterial performance.

This enhanced activity can be attributed to the presence of the citrate layer, which prevents particle aggregation and improves dispersion in aqueous media, thereby increasing the effective contact surface between the nanoparticles and bacterial cells. Indeed, the influence of nanoparticle size and agglomeration on antimicrobial activity has been reported for other nanostructured inorganic systems.^45^ Such colloidal stabilization preserves the nanoscale dimensions of the particles and maximizes the generation of reactive oxygen species (ROS) at the particle-cell interface, which is considered a key mechanism underlying the antimicrobial activity of CeO_2_-based nanomaterials.^46^

When incorporated into the 3D system, however, distinct behaviors were observed for the two bacterial strains. Unlike *S. aureus*, which maintained a dose-dependent response for both GDC and GDC-cit formulations, *E. coli* exhibited the opposite trend in the GDC-cit-containing 3D systems (Figure 8d), where higher nanoparticle concentrations resulted in reduced antibacterial efficacy against the Gram-negative strain. This observation further supports the hypothesis that excessive nanoparticle loading within the polymeric matrix negatively affects the accessibility of reactive surface sites, thereby limiting ROS-mediated antibacterial activity. The stronger effect observed for *E. coli* may additionally be related to the structural complexity of Gram-negative bacteria, whose outer lipid membrane acts as an additional permeability barrier that restricts the interaction between bacterial cells and reactive oxygen species generated at the.

Nevertheless, embedding the nanoparticles within the polymeric platform did not hinder their bactericidal activity; on the contrary, it enhanced their antimicrobial effect since lower nanoparticle concentrations were sufficient to achieve comparable or even higher inhibition than that observed for free nanoparticles in suspension. Importantly, the antimicrobial assays performed using the 3D systems provide indirect evidence that active nanoparticle species remain accessible at the material–medium interface, supporting a surface-mediated mechanism of action rather than a purely diffusion-controlled release process.

This behavior further supports the hypothesis that the antibacterial activity of CeO_2_-based systems is predominantly governed by ROS-mediated mechanisms. In this process, species such as superoxide anions ( $•O_{2}^{-})$ , hydrogen peroxide (H_2_O_2_), and hydroxyl radicals (•OH) induce oxidative stress, resulting in lipid peroxidation, protein oxidation, and DNA damage in microbial cells. Consequently, any reduction in accessible surface area caused by nanoparticle agglomeration is expected to directly compromise ROS generation and, therefore, antibacterial efficiency.

This mechanism resembles the photodynamic inactivation (PDI) process discussed by Hutomo et al.,^47^ in which Cu_2_O-based quantum dots embedded into a PAN/PCL polymeric nanofiber matrix eliminated more than 98% of resistant bacteria within just 30 s under visible-light irradiation due to synergistic ROS generation. Similarly, in the present system, the 3D-printed PVA matrix promotes homogeneous nanoparticle dispersion while preserving their redox activity, thereby maximizing the effective surface area and enhancing the interaction of ROS with microbial targets. This structural organization likely contributes to improved antibacterial performance by facilitating more efficient ROS-mediated cellular damage.

When the antibacterial performance of the 3D-printed systems was compared between bacterial strains, *S. aureus* appeared to be more susceptible than *E. coli* at comparable nanoparticle loadings. This was evidenced by the lower residual viable counts observed for *S. aureus*, particularly in the GDC-cit-containing 3D systems. Although both strains showed substantial reductions in colony-forming units relative to the positive control, the reduction was generally more pronounced for *S. aureus*, corresponding to an estimated reduction of approximately 5.3 log cycles, equivalent to about 99.9995% reduction in the viable bacterial count.

This disparity in antibacterial efficacy can be explained by the intrinsic structural differences between Gram-positive and Gram-negative bacteria. *E. coli* possesses a complex lipid outer membrane that acts as an additional selective barrier, hindering both the diffusion of ROS and the direct interaction between bacterial cells and oxygen vacancies on the ceria surface. In contrast, although *S. aureus* contains a thicker peptidoglycan layer, it lacks this outer lipid membrane and presents a more porous cell wall structure.^48^

To compare the performance of the suspensions and 3D-printed systems, the antibacterial effect should be assessed based on residual CFU counts and log reductions relative to the positive control (Figure 8). In suspension, GDC-cit showed superior antibacterial efficiency. For example, 13.5 mg/mL of GDC-cit achieved an estimated 5.5 log reduction, corresponding to approximately 99.9997% reduction in cultivable bacterial load against *E. coli*, whereas nonfunctionalized GDC required 50 mg/mL to reach a similar effect. After incorporation into the 3D-printed structures, antibacterial activity remained high, with reductions generally above 4 log cycles (93–99% killing), although lower than in suspension. Notably, the lowest GDC-cit concentration in the 3D system (0.83 mg/mL) produced the highest antibacterial effect (5.3 log cycles, 99.2% killing), suggesting that nanoparticle aggregation occurred at higher loadings within the polymer matrix.

Against *S. aureus*, both formulations achieved more than 99.99% reduction in cultivable bacterial load in suspension, although GDC-cit reached this level at a 4-fold lower concentration than uncoated GDC. In the 3D system, GDC-cit retained approximately 90% of its antibacterial activity, maintaining reductions in viable bacterial counts above 99.98%. The less pronounced dose–response behavior observed in the 3D structures is consistent with diffusion-limited access to active nanoparticle sites. Importantly, the preservation of >99% reduction in bacterial counts after fused deposition modeling (FDM) demonstrates that the printing process does not significantly compromise nanoparticle activity or surface-active sites.

Overall, these results establish a direct relationship between the suspension and 3D systems, indicating that the differences in antibacterial performance arise mainly from physical accessibility effects rather than a loss of intrinsic antimicrobial activity. These findings reinforce the potential of the developed materials for structured antimicrobial and topical biomedical applications.

Figure 9a and b show embryotoxicity tests performed with GDC and GDC-cit formulations, respectively. Panels (c) and (d) present photodocumentation of nanoparticle clusters deposited at the bottom of the exposure wells, while SEM images (e–g) illustrate embryos exposed to the nanoparticles. At the highest concentrations tested for both GDC and GDC-cit, nanoparticle adhesion to the chorion was observed (Figure 9d). Agglomerated nanoparticles, indicated by black and white arrows, were consistently found deposited at the bottom of the exposure wells across all test conditions, as corroborated by SEM micrographs (Figure 9e–g). These images reveal the embryo’s surface partially covered by nanoparticles (Figure 9c), the chorion pore with an approximate diameter of 1 μm (Figure 9f), and the nanoparticles appearing as a powder-like material (Figure 9e), presented for comparison with the pore diameter.

**Figure 9:**
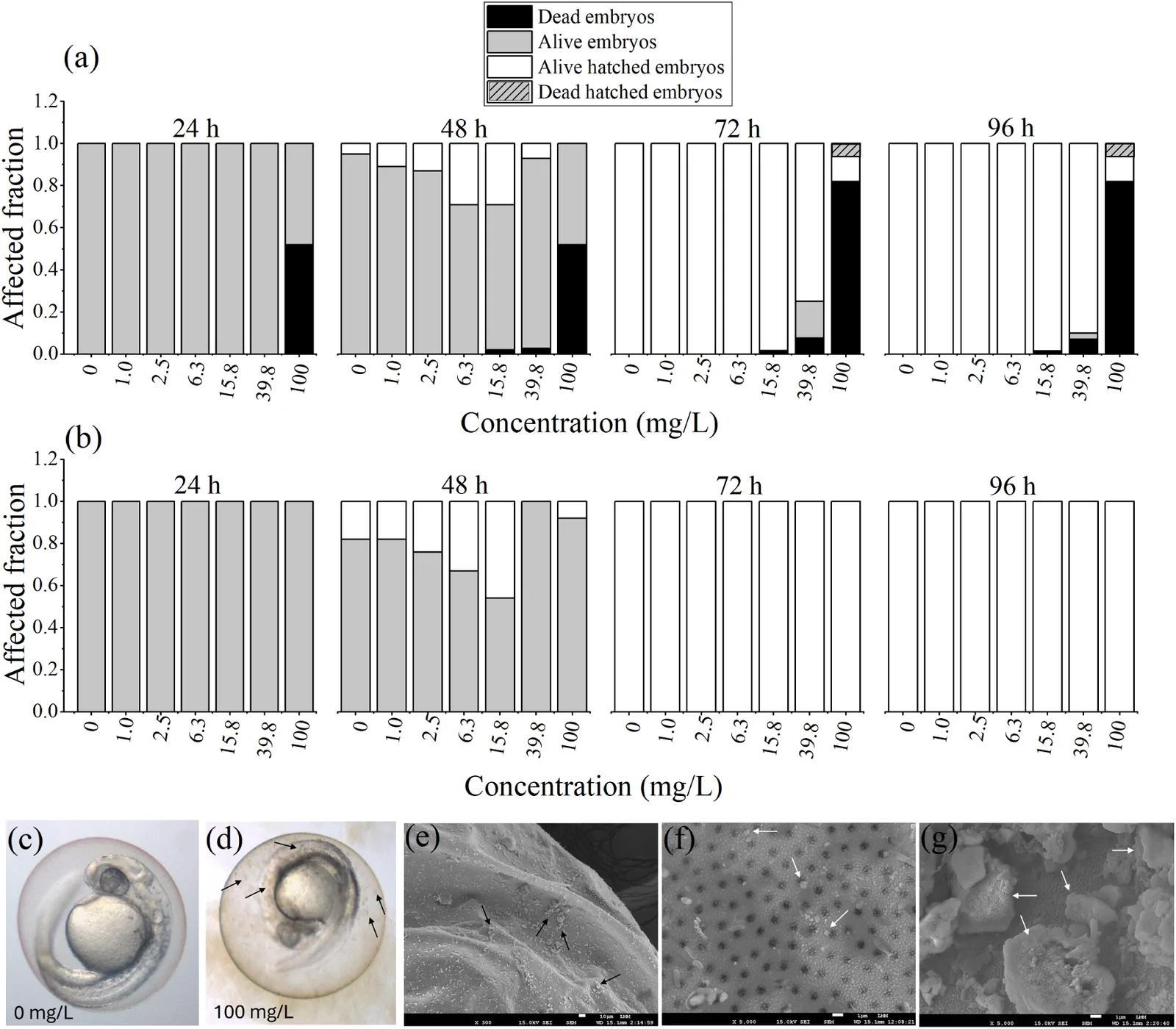
Evaluation of the Fish Embryo Toxicity (FET) assay for GDC and GDC-cit formulations. (a) Overview of the embryotoxicity test performed with GDC nanoparticles and (b) with GDC-cit nanoparticles. Photodocumentation of nanoparticle clusters deposited on the 96-well plate for (c) GDC and (d) GDC-cit (black and white arrows indicate agglomerates). Panels (e–g) show representative SEM images of embryos exposed to GDC and GDC-cit nanoparticles, revealing similar morphological patterns in embryos subjected to both nanoparticle formulations. A total of 60 embryos were analyzed per treatment group (*n* = 60) distributed into three biological replicates. Statistical differences in mortality were evaluated using the Mantel-Cox test.

Similarly, exposure to the highest concentration of 100 mg/L of the GDC formulation resulted in approximately 80% mortality of zebrafish embryos. In contrast, no significant mortality was observed in embryos exposed to the citrate-coated GDC formulation at the same concentration (Figure 9 b) – (LC_50_ > 100 mg/L, NOEC = 100 mg/L).

To provide a more robust quantitative assessment of embryotoxicity, the mortality data obtained for zebrafish embryos exposed to uncoated GDC nanoparticles were additionally analyzed using a nonlinear dose–response regression model with variable slope (Hill equation), performed in GraphPad Prism. During the fitting procedure, the bottom and top parameters were constrained to 0 and 100, respectively, and the quality of the adjustment was evaluated using the coefficient of determination (R^2^). As shown in Figure 10, the uncoated nanoparticles (GDC) exhibited a clear concentration-dependent increase in embryo mortality with an excellent fit to the sigmoidal model (R^2^ = 0.9865).

**Figure 10:**
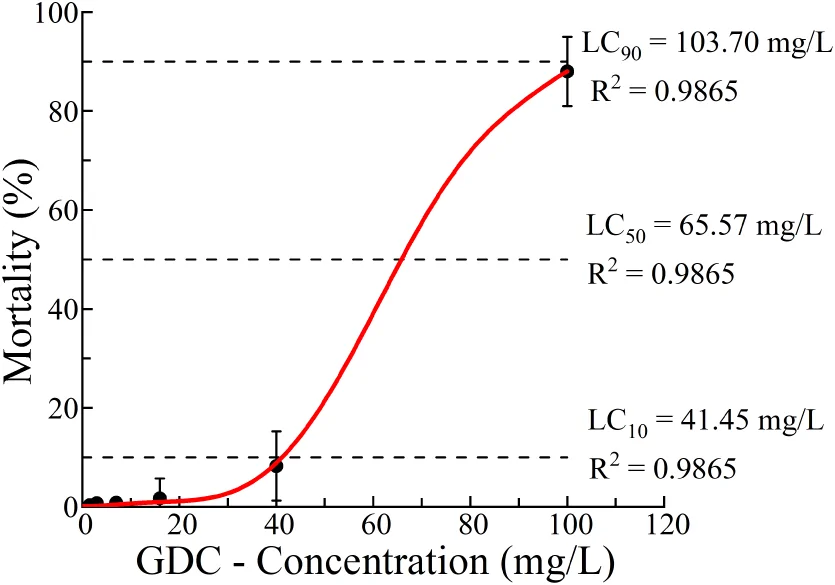
Dose–response curve of acute toxicity of uncoated GDC nanoparticles in zebrafish (*Danio rerio*) embryos.

Based on the sigmoidal model (R^2^ = 0.9865, Figure 10), we determined the following toxicological endpoints for GDC nanoparticles (LC_10_: 41.45 mg/L; LC_50_: 65.57 mg/L; 95% CI: 60.46 to 71.48 mg/L; LC_90_: 103.7 mg/L; and NOEC: 39.8 mg/L).

The determination of LC10 and LC90 for the uncoated group highlights a steep dose–response relationship, with a Hill slope of 4.792. This rapid transition from low to high mortality suggests a threshold effect, likely associated with a critical reduction in colloidal stability and the subsequent sedimentation of uncoated particles at concentrations above 40 mg/L.

This behavior is likely associated with the progressive reduction in colloidal stability at higher concentrations, which may promote particle agglomeration and sedimentation, thereby increasing local nanoparticle exposure to the embryos. In contrast, citrate-coated GDC nanoparticles did not induce significant lethality up to the highest tested concentration, resulting in LC_50_ values above 100 mg/L and a NOEC of 100 mg/L. This outcome suggests that the citrate coating significantly reduced the nanoparticle–embryo interaction, most likely by increasing colloidal stability and preventing aggregation in the exposure medium. Improved dispersion limits direct contact between nanoparticles and embryonic membranes, thereby mitigating acute embryotoxicity and enhancing the overall safety profile of the formulations. This phenomenon is consistent with previous studies highlighting the protective role of surface functionalization in reducing nanotoxicity.^49,50^

These observations are consistent with previous studies reported in the literature. For example, Correia et al. (2020) evaluated the effects of uncoated GDC NPs using the fish *Oncorhynchus mykiss* as a model organism and reported alterations in enzymatic biomarkers and histopathological changes in organisms exposed to the highest concentration (25 mg/L).^51^ Similarly, Pecoraro et al. investigated the toxicity of uncoated nanoparticles in zebrafish embryos and observed that the lethal concentration exceeded the highest tested dose (100 mg/L), although alterations in biomarkers such as ethoxyresorufin-O-deethylase (EROD) activity were detected.^52^ More recently, Liu et al. reported comparable toxicological effects when exposing zebrafish embryos to cerium-based nanoparticles, observing low acute lethality but measurable changes in gene expression profiles.^53^

Although the present demonstration shows low acute toxicity for citrate-functionalized systems and suggests limited risk of gadolinium release under the investigated conditions, it is important to acknowledge that long-term stability, potential ion leaching, and *in vivo* safety remain critical aspects requiring further investigation. These considerations are particularly relevant for future biomedical or environmental applications and are highlighted as directions for future work.

It is also important to clarify that the concentration ranges employed in the cytotoxicity, antimicrobial, and zebrafish embryo assays were intentionally broad to evaluate concentration-dependent biological responses; they should not be interpreted as direct clinical exposure levels. In practical topical applications, nanoparticle exposure is expected to be localized and modulated by the 3D polymeric matrix, which restricts nanoparticle mobility and controls their accessibility at the material–tissue interface. Consequently, the effective exposure under realistic conditions is likely lower and more spatially confined than the nominal concentrations used in suspension-based *in vitro* assays. Nonetheless, further studies involving nanoparticle release kinetics, skin permeation, and *in vivo* validation remain necessary before clinical translation.

## 4 Conclusions

This study successfully developed a 3D-printed PVA-based nanocomposite incorporating Gd-doped CeO_2_ nanoparticles for topical antimicrobial applications. The nanoparticles were efficiently synthesized and integrated into PVA filaments via hot-melt extrusion, enabling the fabrication of stable, uniform structures by fused deposition modeling without compromising the crystalline integrity of the inorganic phase.

The physicochemical analyses revealed that citrate functionalization plays a central role in controlling nanoparticle dispersion and interfacial behavior. Dynamic light scattering and zeta potential measurements demonstrated a substantial improvement in colloidal stability, with the hydrodynamic diameter decreasing from ∼600 nm to ∼250 nm and the surface potential shifting from approximately −15 mV to −35 mV after citrate coating. Complementary XRD, FTIR, and Raman analyses confirmed that citrate functionalization enhances nanoparticle–polymer compatibility, promotes stronger interfacial interactions, and partially passivates surface oxygen vacancies while maintaining the fluorite crystal structure after extrusion and 3D printing.

From a biological perspective, citrate-coated nanoparticles exhibited more predictable dose-dependent behavior, improved colloidal stability, and superior antimicrobial activity compared to uncoated systems.

In suspension, GDC-cit nanoparticles achieved approximately 5.5-log bacterial reduction against *E. coli* at concentrations nearly four times lower than uncoated nanoparticles. Against *S. aureus*, both systems produced reductions above 99.99%, although citrate-functionalized nanoparticles maintained equivalent efficacy at significantly lower concentrations. Importantly, incorporation into the 3D-printed matrices preserved most of the antimicrobial performance, with antibacterial reductions generally remaining above 4 log cycles and, in some conditions, exceeding 5-log reduction even at low nanoparticle concentrations. These findings demonstrate that the polymeric matrix does not suppress the intrinsic antimicrobial activity of the nanoparticles but rather modulates their accessibility and dispersion.

The toxicological evaluations further emphasized the importance of surface engineering. While uncoated nanoparticles induced approximately 80% zebrafish embryo mortality at 100 mg/L, citrate-coated systems exhibited no significant mortality at the same concentration, indicating markedly improved biocompatibility. Similarly, HaCaT keratinocyte assays showed that citrate-functionalized nanoparticles produced more reproducible and controlled cellular responses, supporting the role of colloidal stabilization in improving the biological performance.

Overall, this work establishes a direct quantitative relationship between nanoparticle behavior in suspension and in 3D-printed systems, demonstrating that the observed differences in antimicrobial performance are governed primarily by nanoparticle accessibility and dispersion rather than by a loss of intrinsic activity during processing. The results highlight the potential of citrate-functionalized GDC nanoparticles as active nanofillers for structurally controlled antimicrobial platforms and provide important advances toward the development of customizable 3D-printed biomedical materials. Nevertheless, despite the promising results, this study represents an early-stage material and biological evaluation of the proposed platform. Important aspects required for translational application, including long-term storage stability, nanoparticle release behavior, skin permeation, advanced infection models, and *in vivo* topical validation, remain to be systematically investigated. Furthermore, additional studies addressing long-term biocompatibility and functional stability under practical-use conditions will be necessary. Future work will focus on these aspects to further advance the translational potential of the developed platform.
